# AlphaFold accelerated discovery of psychotropic agonists targeting the trace amine–associated receptor 1

**DOI:** 10.1126/sciadv.adn1524

**Published:** 2024-08-07

**Authors:** Alejandro Díaz-Holguín, Marcus Saarinen, Duc Duy Vo, Andrea Sturchio, Niclas Branzell, Israel Cabeza de Vaca, Huabin Hu, Núria Mitjavila-Domènech, Annika Lindqvist, Pawel Baranczewski, Mark J. Millan, Yunting Yang, Jens Carlsson, Per Svenningsson

**Affiliations:** ^1^ Science for Life Laboratory, Department of Cell and Molecular Biology, Uppsala University, Box 596, SE-751 24 Uppsala, Sweden.; ^2^ Neuro Svenningsson, Department of Clinical Neuroscience, Karolinska Institute, SE-171 76 Stockholm, Sweden.; ^3^ Department of Neurology, James J. and Joan A. Gardner Family Center for Parkinson's Disease and Movement Disorders, University of Cincinnati, Cincinnati, OH, USA.; ^4^ Department of Pharmacy, SciLifeLab Drug Discovery and Development Platform, Uppsala University, Box 580, SE-751 23 Uppsala, Sweden.; ^5^ Neuroinflammation Therapeutic Area, Institut de Recherches Servier, Centre de Recherches de Croissy, Paris, France and Institute of Neuroscience and Psychology, College of Medicine, Vet and Life Sciences, Glasgow University, Scotland, Glasgow, UK.

## Abstract

Artificial intelligence is revolutionizing protein structure prediction, providing unprecedented opportunities for drug design. To assess the potential impact on ligand discovery, we compared virtual screens using protein structures generated by the AlphaFold machine learning method and traditional homology modeling. More than 16 million compounds were docked to models of the trace amine–associated receptor 1 (TAAR1), a G protein–coupled receptor of unknown structure and target for treating neuropsychiatric disorders. Sets of 30 and 32 highly ranked compounds from the AlphaFold and homology model screens, respectively, were experimentally evaluated. Of these, 25 were TAAR1 agonists with potencies ranging from 12 to 0.03 μM. The AlphaFold screen yielded a more than twofold higher hit rate (60%) than the homology model and discovered the most potent agonists. A TAAR1 agonist with a promising selectivity profile and drug-like properties showed physiological and antipsychotic-like effects in wild-type but not in TAAR1 knockout mice. These results demonstrate that AlphaFold structures can accelerate drug discovery.

## Teaser

AlphaFold models outperform traditional methods in virtual screening and identify leads for the development of antipsychotics.

## INTRODUCTION

Recent advances in machine learning have enabled breakthroughs in protein structure prediction. Specifically, AlphaFold from DeepMind has been demonstrated to predict protein structures from sequence with near-experimental accuracy and outperformed traditional techniques in community wide assessments (*1*, *2*). AlphaFold structures for the entire human proteome were recently made available, which provided access to models of numerous therapeutically relevant proteins (*3*). This expansion of structural coverage has led to increasing interest in using AlphaFold models for drug design.

Access to atomic resolution structures of a target protein can accelerate the drug discovery process by facilitating hit identification and guiding compound optimization (*4*–*6*). However, structure determination remains challenging for many therapeutically relevant proteins. Among these are the family of the G protein–coupled receptors (GPCRs), which play important roles in physiological processes and are the targets of >34% of Food and Drug Administration–approved drugs (*7*). As rational drug design using crystal and cryo–electron microscopy (cryo-EM) structures of GPCRs has proven to be efficient (*6*, *8*–*10*), AlphaFold has sparked interest in exploiting computational models in ligand discovery campaigns for the many receptors of unknown structure. However, several studies have questioned whether AlphaFold models can be used to predict the structures of GPCR-drug complexes (*11*–*13*). Recently, Karelina *et al.* (*11*) demonstrated that AlphaFold can model GPCR binding site structures with high accuracy. In contrast, computational docking to these AlphaFold models resulted in substantially less accurate ligand binding modes than obtained using experimentally determined GPCR structures. Although AlphaFold models of GPCR binding sites were considerably better than homology models, there was no notable difference in accuracy between ligand binding modes predicted using these two types of computational models. Evaluations of virtual screening performance reached similar conclusions, indicating that AlphaFold models need to be further optimized for drug design applications. In several studies, molecular docking to AlphaFold models did not enrich known ligands as well as experimentally determined structures of protein-ligand complexes (*14*, *15*). Together, these retrospective assessments have suggested that AlphaFold is not suitable for structure-based drug design applications and may not improve models of protein-ligand complexes beyond traditional template–based methods.

In this work, we further investigated the utility of AlphaFold models in structure-based virtual screening and compared the performance of AlphaFold to traditional homology modeling. In contrast to previous studies, prospective docking screens were carried out and experimental evaluation of top-ranked compounds highlighted differences between the two structure prediction methods. The trace amine–associated receptor 1 (TAAR1), a GPCR for which no experimental structure was available at the time of the study, was selected as the target of the virtual screen. TAAR1 belongs to the class A (Rhodopsin-like) family of GPCRs and is activated by a variety of trace amines including tyramine, β-phenethylamine (β-PEA), and catecholamine metabolites such as 3-methoxytyramine (*16*, *17*). TAAR1 has garnered substantial clinical interest over the last decade as a potential therapeutic target for several neuropsychiatric disorders, particularly schizophrenia (*17*, *18*). Activation of TAAR1 in dopaminergic, serotonergic, and glutamatergic neurons appears to have an overall inhibitory effect on cell firing, and complementary behavioral observations suggest that agonists could be effective in the treatment of drug addiction, bipolar disorder, and schizophrenia (*18*, *19*). Two TAAR1 agonists, Ulotaront (Sunovion) and Ralmitaront (Hoffmann–La Roche), already advanced into clinical trials for the treatment of several conditions including narcolepsy (*20*), psychosis in Parkinson’s disease (*21*, *22*), and schizophrenia (*23*).

We first predicted the structure of TAAR1 using both homology modeling and AlphaFold. A library of 16 million compounds was then docked to each set of models, followed by experimental evaluation of top-ranked molecules. Both virtual screens identified TAAR1 ligands, but, in contrast to previous observations, the AlphaFold model performed considerably better than the homology model. From the AlphaFold docking screen, we selected 30 highly ranked compounds, and 18 (60%) of these were confirmed to be TAAR1 agonists, which was a more than twofold higher hit rate than for the homology model. Several hits showed nanomolar potency and exploration of structure-activity relationships, selectivity, and pharmacokinetic properties led to the selection of a candidate for in vivo evaluation. This lead compound regulated body temperature and displayed antipsychotic-like effects in wild-type but not in TAAR1 knockout mice. Our work demonstrates how AlphaFold models can be used successfully in structure-based virtual screening campaigns to identify leads for the development of antipsychotics.

## RESULTS

### Comparison of predicted TAAR1 structures

Predictions of the TAAR1 structure by AlphaFold and homology modeling were assessed by comparing the binding site models and performing molecular docking calculations of known agonists using DOCK3.7 (*24*). Structures representing four aminergic GPCRs in an active conformation were first evaluated as templates for homology modeling (table S1). The sequence identity (33 to 37%) was high enough to expect a good performance by GPCR homology modeling (*25*), and the templates represented diverse orthosteric sites that bind monoamine neurotransmitters. The ability of homology models to enrich known ligands over decoys has been demonstrated to reflect the accuracy of GPCR structures (*26*–*28*) and was assessed on the basis of docking calculations of 173 previously identified TAAR1 ligands (*22*, *29*) and 11,392 property-matched decoys (non-binders) (*30*) to 250 models per template. We quantified ligand enrichment using the adjusted logarithm of the area under the curve (LogAUC), and positive values of this metric indicate that docking performs better than random selection (*31*). Among the four templates, we selected the β_1_ adrenergic receptor for further evaluation because this structure led to the best maximal and average ligand enrichment (table S1). The virtual screening performance of homology modeling for this template was then optimized by increasing the number of generated structures to 1000. The best performing models (98th percentile) had a median LogAUC value of 25 and enriched the known TAAR1 agonist by 11-fold over what is expected at random among the top-ranking 1% of the docked database (EF1%).

**Fig. 1.**
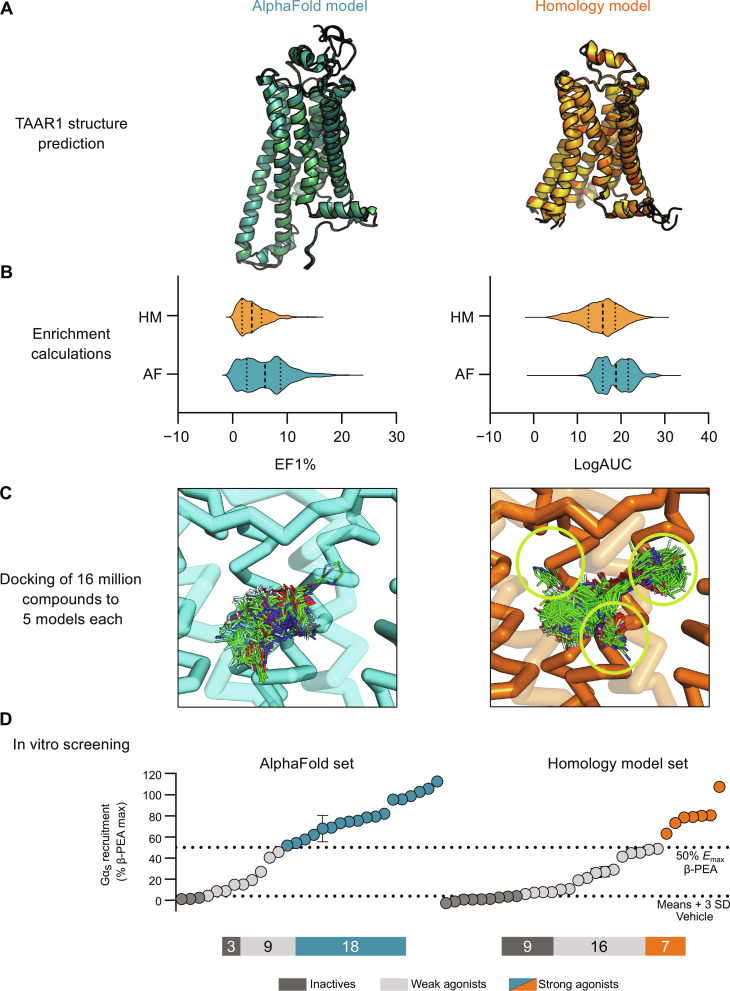
Virtual screening performance of homology and AlphaFold models. (**A**) TAAR1 models were generated using the homology modeling and AlphaFold methods. (**B**) Ligand enrichment based on docking calculations of known TAAR1 ligands and decoys for 1000 models generated by homology modeling (HM) and AlphaFold (AF). Distributions of LogAUC and EF1% values are represented as violin plots with the median shown as a dashed line. (**C**) A library of 16 million compounds was docked to ensembles of five homology and AlphaFold models. Binding modes of top-ranked compounds from the two screens illustrate differences between the AlphaFold and homology models, which are shown as cyan and orange ribbons, respectively. Docked compounds are shown as lines and occupy a larger number of subpockets in the homology model, which are marked with green circles. (**D**) Experimental evaluation of 62 compounds predicted by docking screens using the homology (right) and AlphaFold (left) models. Compound activity (20 μM) was evaluated by measuring recruitment of Gα_s_, which was normalized as a percentage of the response elicited by a saturating concentration of β-PEA. Compounds inducing more than 50% response are shown as orange/teal circles (homology modeling/AlphaFold), significant but less than 50% response as light gray circles, and insignificant response as dark gray circles. Data represent means ± SD of two to four technical replicates.

Analogous to the assessment of the homology models, machine learning predicted structures of TAAR1 were evaluated by docking the same database of actives and decoys to the orthosteric site. We generated an ensemble of 1000 TAAR1 structures using AlphaFold. The AlphaFold confidence score [predicted local distance difference test (pLDDT)] exceeded 90 for the vast majority of the binding site residues, which indicated that the structure prediction should be expected to be highly accurate (*1*). Whereas the homology modeling protocol resulted in an active-like receptor structure due to the template selection, the AlphaFold models represented an overall inactive conformation of TAAR1. The ligand enrichment of the AlphaFold structures was considerably better than the result obtained for the homology models (median LogAUC = 28 for 98th percentile) and the median EF1% was 18 (Fig. 1).

The homology and AlphaFold models were further compared by analyzing the binding site structure and predicted receptor-agonist complexes. We selected a set of five diverse binding site structures among the models that showed the best ligand enrichment. All the five AlphaFold structures performed better than the homology models (table S2), which reflected the overall better enrichment of these models compared to the homology models. The binding mode of β-PEA in the models was overall similar in the structures predicted by homology modeling and AlphaFold, and the agonist formed interactions observed in experimentally determined structures of other aminergic GPCRs (fig. S1) (*32*). For example, β-PEA formed a salt bridge to D103^3.32^ (superscripts represent Ballesteros-Weinstein numbering) (*33*, *34*), and the aromatic ring of this agonist interacted with a deeply buried hydrophobic pocket created by transmembrane helices (TM) 3, 5, and 6. The main difference between the homology model and AlphaFold binding sites was the size of the pocket. This was due to distinct structural differences in the extracellular TM region, the second extracellular loop (EL2), and side-chain conformations that together led to a more compact AlphaFold pocket. These differences created additional subpockets in the binding site of the homology models that were not present in the structures predicted by AlphaFold.

### Docking for TAAR1 ligands

To evaluate whether the AlphaFold and homology models could guide the discovery of TAAR1 ligands, two structure-based virtual screens of 16 million commercially available compounds were performed. The chemical library was composed of fragment-like compounds (molecular weight < 250 Da) from the ZINC database (*35*), which contained diverse molecules of a size similar to the endogenous TAAR1 agonists. In each screen, we used the five selected receptor models that performed well in enriching known TAAR1 ligands in retrospective docking calculations. By using an ensemble of receptor structures, we anticipated that the virtual screening results would be less dependent on the model selection and thereby reflect the performance of the AlphaFold and homology modeling predictions of the binding site. Moreover, as the receptor structure was held rigid in the docking calculations, our ensemble docking approach partially accounted for binding site flexibility.

The two docking screens evaluated over 218 trillion complexes in total, and an average of 6.8 million and 11.3 million were successfully docked to the AlphaFold and homology models, respectively. The difference between the number of successfully docked compounds reflected the smaller size of the TAAR1 binding pocket predicted by AlphaFold (Fig. 1). For each screen, compounds were ranked on the basis of the best docking score among the five models, and top-ranked molecules were further processed to identify candidates for experimental testing, e.g., by excluding compounds similar to known TAAR1 ligands and with chemical motifs revealed by pan-assay interference compounds (PAINS) (*36*). The remaining top-ranked molecules were clustered by topological similarity to identify a diverse set of candidates for experimental testing. From the 2000 top-ranking clusters, we selected a set of 30 compounds based on the AlphaFold model, and 32 compounds were prioritized from the homology modeling screen (table S3, A and B). In the selection of compounds for experimental evaluation, we focused on identifying molecules that formed key interactions observed in structures of other aminergic GPCRs (*32*). For example, the selected compounds typically formed a salt bridge with D103^3.32^, and their aromatic groups interacted with the hydrophobic pocket formed by TM3, TM5, and TM6. In addition, we took into account that several energy terms are either poorly described by the docking scoring function (e.g., desolvation penalties for unsatisfied hydrogen bond donors and acceptors) or not considered at all (e.g., ligand strain and receptor desolvation), as described previously (*37*). Notably, the structural differences between the AlphaFold and homology models led to the enrichment of diverse compounds with contrasting shapes and sizes. Only 16% of the top 10,000 compounds from the two virtual screens overlapped. This result can be compared to the average percentage of compounds that overlapped between structures generated by the same method, which was two- to fourfold higher (59 and 30% for the AlphaFold and homology models, respectively). Because of the larger and more open binding site of the TAAR1 homology models, top-ranked compounds from this docking screen were generally larger and represented more diverse chemical structures (Fig. 1 and fig. S2).

**Fig. 2.**
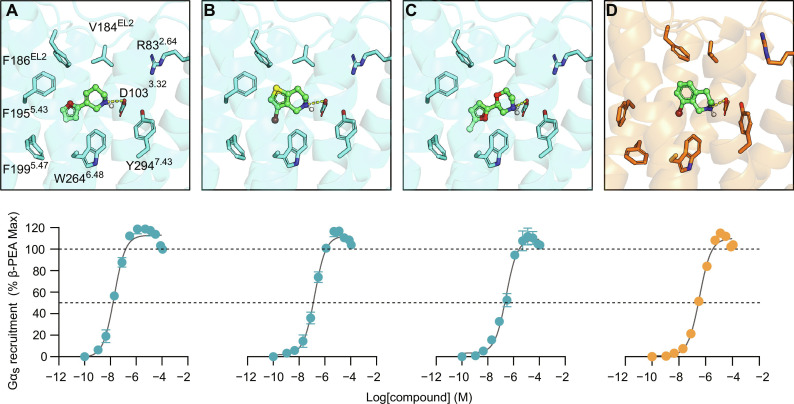
Binding modes and concentration-response curves of discovered agonists. (**A** to **C**) Predicted binding modes of the three most potent agonists (**30**, **29**, and **27**, respectively) identified by docking to the AlphaFold model (magenta cartoons). (**D**) Predicted binding mode of most potent agonist (compound **62**) identified by docking to the homology model (orange cartoons). Ligands and key binding site residues are shown as sticks. The corresponding concentration-response curve from the G protein recruitment assay is shown below each receptor-ligand complex. Data represent means ± SEM of three independent experiments.

The 62 predicted ligands were purchased from commercial vendors and tested for activity at a concentration of 20 μM in a luminescence complementation–based assay measuring recruitment of mini-G_s_ to the human TAAR1 (*38*). A set of 25 compounds displayed agonist activity and an efficacy greater than 50% of the maximal effect of β-PEA (Fig. 1). Unlike the TAAR1 reference antagonist RTI-7470-44 (*39*), none of the compounds antagonized the effect of β-PEA on TAAR1 (fig. S3). As a control for artifactual assay activity, the 62 compounds were also evaluated at another G_s_-coupled GPCR [the A_2A_ adenosine receptor (A_2A_R), which does not belong to the group of aminergic receptors] using the same assay, and no agonist or antagonist activity was observed (fig. S4, A to C). These results showed that the 25 hits from the docking screens specifically activate TAAR1 rather than causing assay interference or unspecific signal amplification. Concentration-response experiments showed that the compounds were agonists of TAAR1 with negative logarithm of the half maximal effective concentration (pEC_50_) values ranging from 4.9 to 7.5 [maximum effect (*E*_max_) = 52 to 112% of the maximal effect of β-PEA]. Among these, 18 agonists were identified based on the AlphaFold models of TAAR1, corresponding to a hit rate of 60%, and had pEC_50_ values ranging from 5.1 to 7.5 (compounds **13** to **30**, Table 1). The hit rate from the homology modeling screen was 22% (seven compounds: **56** to **62**, Table 2), and the pEC_50_ values of these agonists ranged from 4.9 to 6.5. The hit rate achieved with the AlphaFold model was hence more than twofold higher, and the three most potent compounds identified in this study also emerged from this screen. A more detailed analysis of the docking results showed that five of the experimentally tested compounds were present among the top-ranked 2000 clusters of both screens. However, even if the hit rate was adjusted for this overlap, the AlphaFold model (54% adjusted hit rate) performed better than the homology model (22% adjusted hit rate). We also analyzed the impact of including a larger number of top-ranked compounds in the calculation of hit rates. If a 10-fold larger number of compounds were considered, then the hit rate of the AlphaFold (50% hit rate) screen remained higher than that based on the homology model (33% hit rate). The vast majority of the identified agonists were dissimilar to previously identified ligands [Tanimoto similarity coefficient (*T*_c_) < 0.35 to known TAAR1 agonists from the ChEMBL database] (*29*). The three most potent compounds from the AlphaFold screen (pEC_50_ = 6.6 to 7.5) were predicted to form similar interactions with the binding site and represented diverse scaffolds (compounds **27**, **29**, and **30**, *T*_c_ of 0.13 to 0.20 to known TAAR1 agonists) (Fig. 2, A to C). The most potent agonist from the homology model screen (**62**, pEC_50_ = 6.5) represented a superstructure of β-PEA, and this compound was also top-ranked in the AlphaFold screen (Fig. 2D). The best AlphaFold compound (**30**, pEC_50_ = 7.5) was a full agonist and ~10-fold more potent than the reference agonist β-PEA (pEC_50_ = 6.6). This agonist scaffold was composed of a 3-piperideine connected to a furan ring that has not previously been described as a TAAR1 agonist. Furthermore, despite the relatively simple chemical structure and commercial availability of compound **30**, there were no bioactivity data in the ChEMBL database for this molecule.

**Table 1.** Discovered agonist from the docking screen against AlphaFold models of TAAR1.

ID	Structure	Rank*	pEC_50_	ID	Structure	Rank*	pEC_50_
			(*E*_max_, %)^†^				(E_max_, %)^†^
**β-PEA**	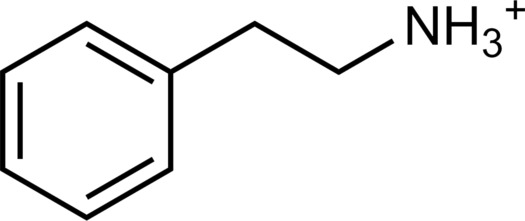	–	6.6(100)	**Ulotaront**	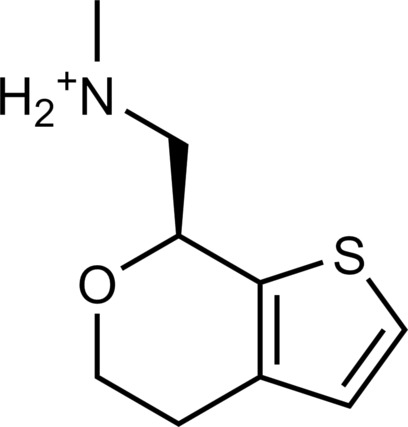	–	6.1^‡^(96.4)
**13**	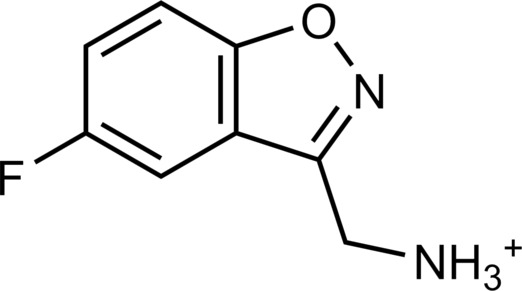	2938(35,310)	5.5(51.8)	**14**	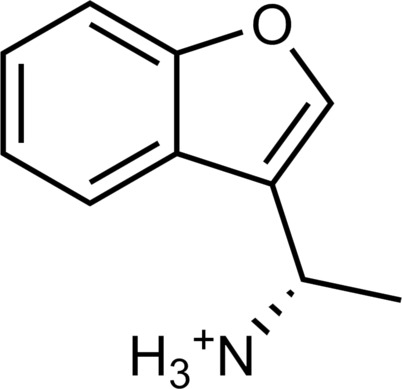	1098(109,532)	5.1(54.3)
**15**	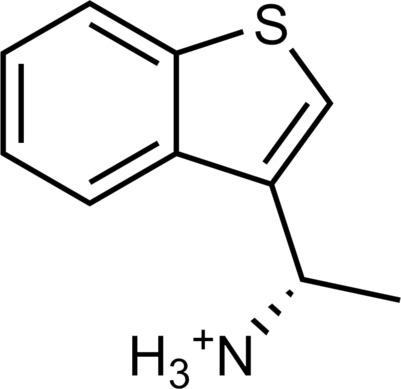	311(56,230)	5.2(57.5)	**16**	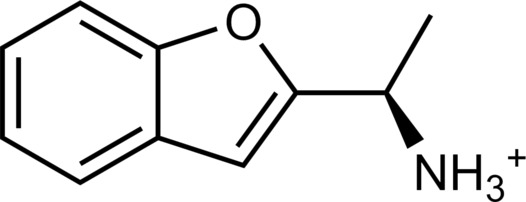	4001(44,811)	5.8(61.9)
**17**	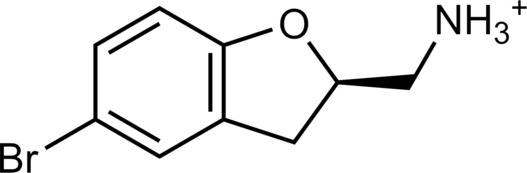	1017(865,757)	5.2(67.8)	**18**	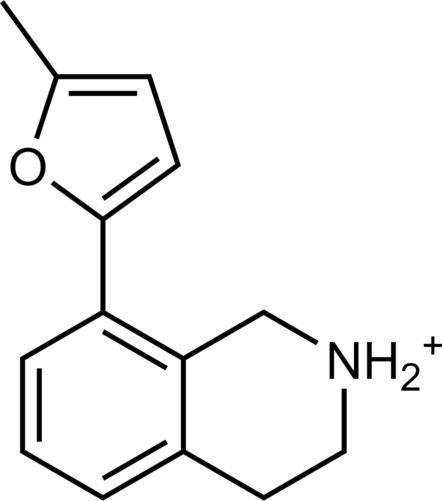	84(437,792)	6.2(68.7)
**19**	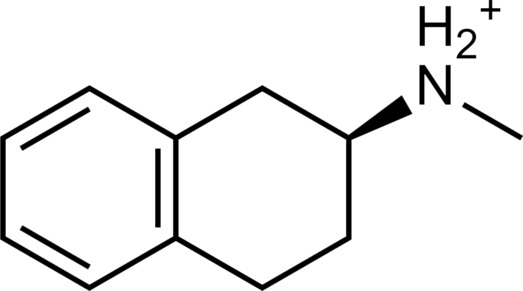	573(382,074)	5.4(74.5)	**20**	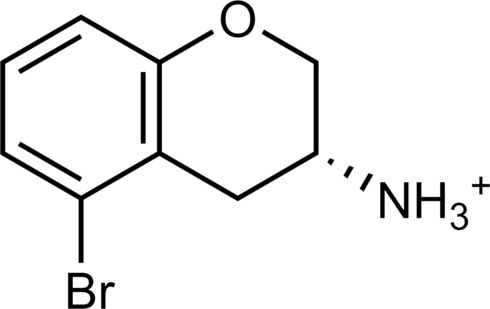	151(25,997)	5.4(74.5)
**21**	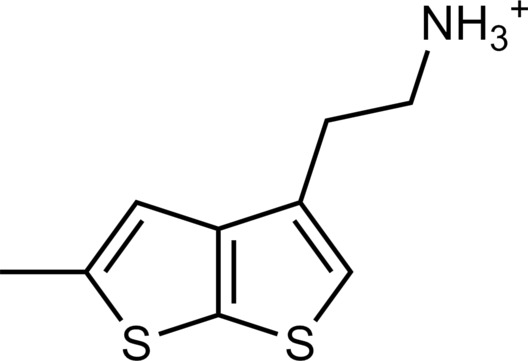	555(195,139)	5.5(75.2)	**22**	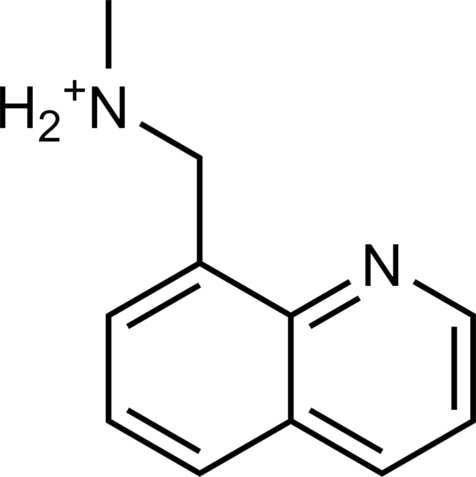	3272(195,139)	5.5(78.5)
**23**	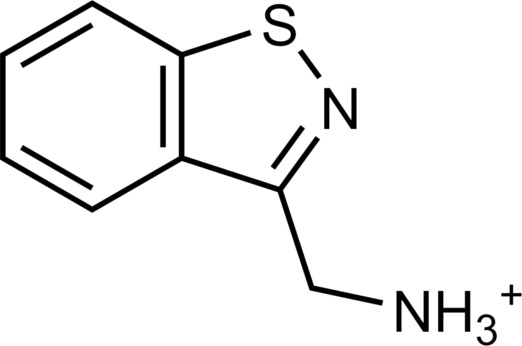	1528(37,423)	5.4(79.2)	**24**	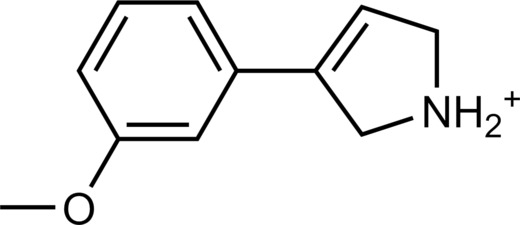	668(18592)	5.8(81.4)
**25**	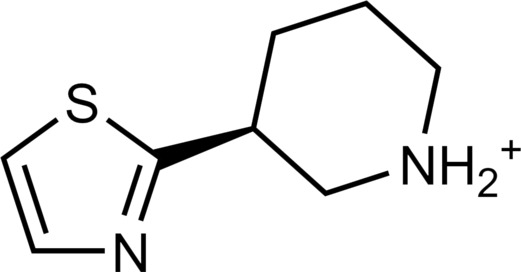	717(85,960)	6.0(95.3)	**26**	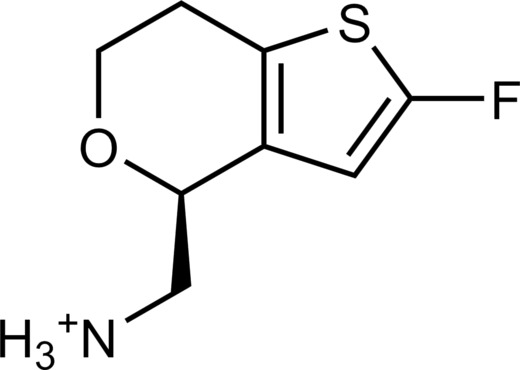	3040(212,223)	6.0(95.5)
**27**	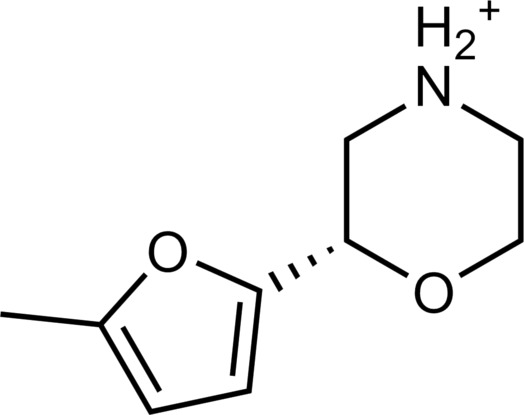	3060(65,537)	6.6(98.7)	**28**	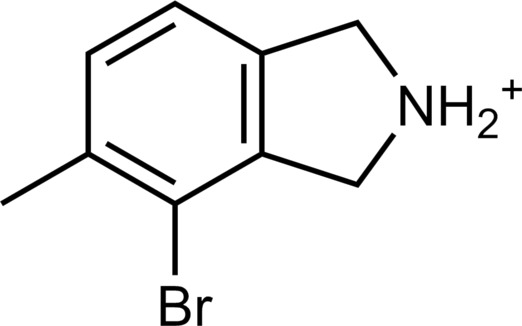	187(22,718)	6.4(102.6)
**29**	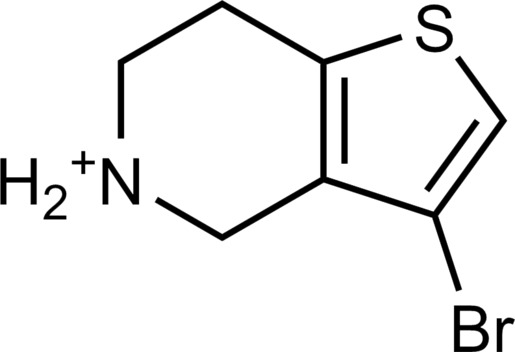	381(7957)	6.8(105.7)	**30**	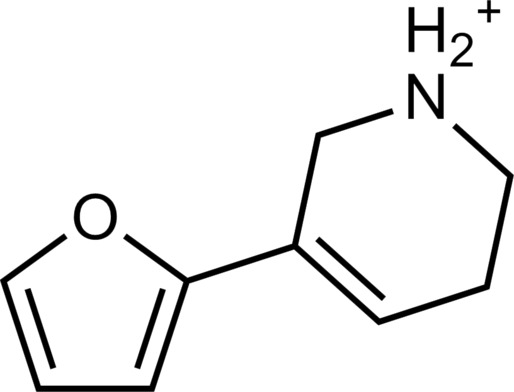	963(22,595)	7.5(112.5)

*The rank in the AlphaFold docking screen before clustering of top-ranked compounds. All the experimentally evaluated compounds were present in the 2000 top-ranking clusters. The rank of the same compound in the screen against the homology models is shown in parentheses. None of the discovered agonists would have been among the 2000 top-ranked clusters in the homology model docking screen.

†pEC_50_ values at TAAR1 using a G protein recruitment assay. *E*_max_ values are relative to the effect of the reference agonist β-PEA.

‡Potency and *E*_max_ values from Saarinen *et al.* (*38*).

**Table 2.** Discovered agonists from the docking screen against homology models of TAAR1. N/A, not applicable.

ID	Structure	Rank*	pEC_50_	ID	Structure	Rank*	pEC_50_
			(*E*_max_, %)^†^				(*E*_max_, %)^†^
**56**	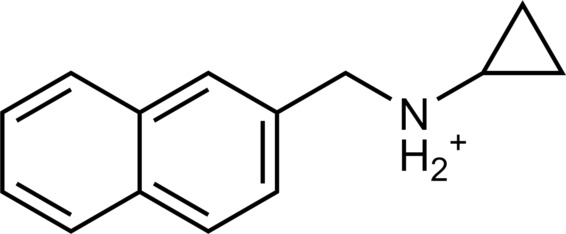	2811(467,247)	4.9(63.0)	**57**	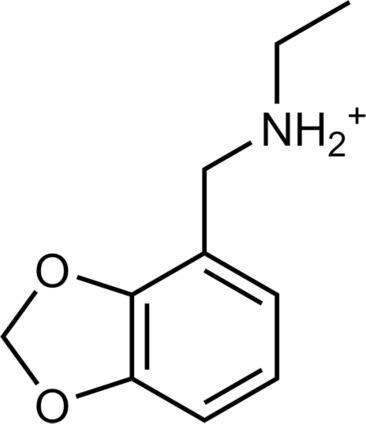	290(118,716)	5.3(73.2)
**58**	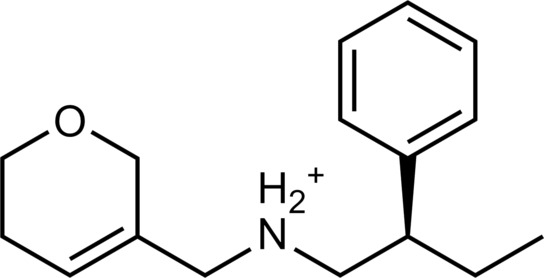	3350(N/A^‡^)	5.1(78.4)	**59**	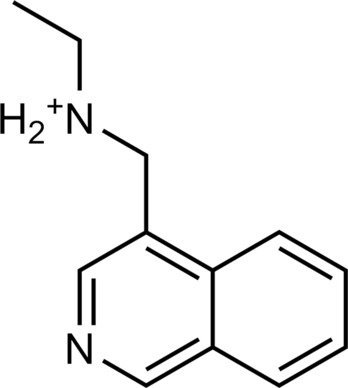	550(12,903)	5.2(78.8)
**60**	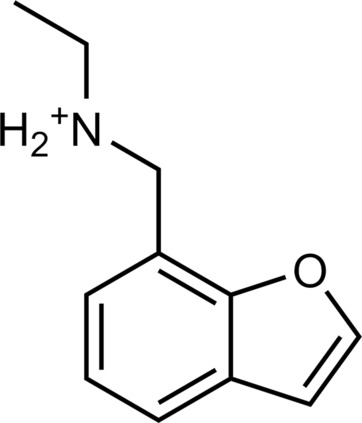	1877(165,473)	5.5(79.9)	**61**	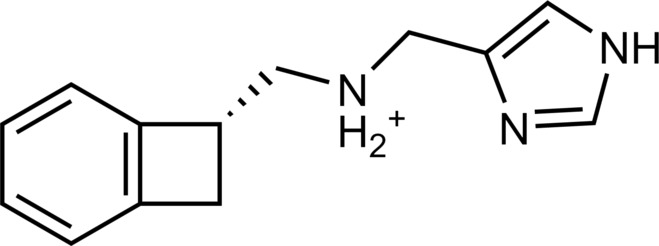	1359(39,818)	5.3(80.3)
**62**	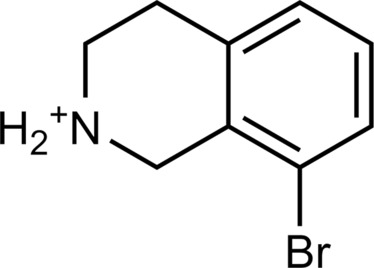	3728(469)	6.5(107.4)				

*The rank in the homology model docking screen before clustering of top-ranked compounds. All the experimentally evaluated compounds were present in the 2000 top-ranking clusters. The rank of the same compound in the screen against the AlphaFold models is shown in parentheses. One of the discovered agonists (**62**) would have been among the top-ranked clusters in the AlphaFold docking screen.

†pEC_50_ values at TAAR1 using a G protein recruitment assay. *E*_max_ values are relative to the effect of the reference agonist β-PEA.

‡Compound failed to dock to the AlphaFold models due to steric clashes.

### Structure-activity relationships of TAAR1 activation

**Fig. 3.**
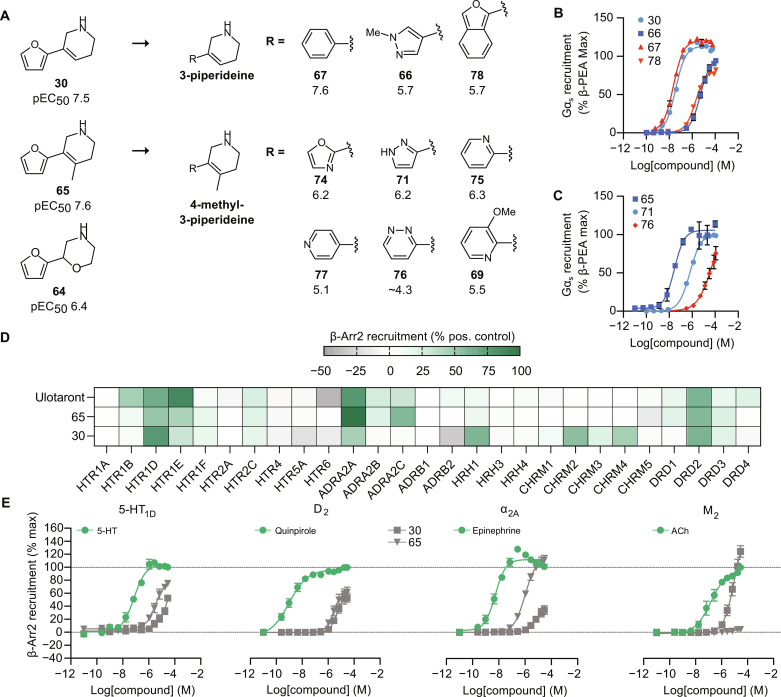
Structure-activity relationships and selectivity profile of TAAR1 agonists. (**A**) Exploration of the structure-activity relationships of the scaffold represented by compound **30**. (**B** and **C**) Concentration-response curves for analogs of compound **30** from the G protein recruitment assay. (**D**) Heatmap of PRESTO-TANGO screening results for 27 aminergic GPCRs using Ulotaront, **65**, and **30**. Data represent three to six technical replicates per receptor. β-Arrestin recruitment was normalized as a percentage of the signal emitted by the positive controls [5-hydroxytryptamine (5-HT), quinpirole, epinephrine, and acetylcholine (Ach)] for each receptor (table S5). (**E**) Concentration-response curves from PRESTO-TANGO assay for compounds **30** and **65** and controls at the 5-HT_1D_, D_2_, α_2A_, and M_2_ receptors. Data represent means ± SEM of three independent experiments.

We selected the most potent compound from the AlphaFold screen (compound **30**, pEC_50_ = 7.5) as a starting point in the evaluation of analogs. Compounds were identified by searching in commercial chemical libraries containing billions of make-on-demand compounds in combination with the generation of a virtual library of molecules that could be synthesized in-house using readily available building blocks. The analogs were docked to the AlphaFold models, and visual inspection of the predicted binding modes led to the selection of 16 compounds (**63** to **78**, table S4). We evaluated this diverse set of compounds with the goal to identify potent agonists with selectivity and pharmacokinetic properties suitable for in vivo studies. All compounds were agonists with pEC_50_ values ranging from 4.3 to 7.6, and they collectively revealed a clear pattern of structure-activity relationships at TAAR1 (Fig. 3A).

The analogs were all predicted to maintain the key interaction with D103^3.32^, and we explored the effect of modifying the moiety forming a salt bridge with this residue. Replacing the 3-piperideine of compound **30** with a morpholino group reduced agonist potency by >10-fold (compound **64**, pEC_50_ = 6.4), whereas a methyl-substituted variant of the 3-piperideine was equipotent (Fig. 3A). We evaluated diverse aromatic groups with varying polarity in the pocket that was occupied by a furan in the predicted TAAR1 complex with compound **30**. Replacing the furan with a benzyl moiety resulted in a potent agonist (compound **67**, pEC_50_ = 7.6). However, further increasing the size or polarity of the aromatic group reduced potency to the micromolar range (Fig. 3A). These results were consistent with the AlphaFold model, which predicted that these agonists were bound in a small and enclosed pocket with primarily hydrophobic side chains. Six agonists in the series of 3-piperideine–based compounds showed potency comparable or superior to Ulotaront, a TAAR1 agonist undergoing clinical trials for several disorders (*20*, *21*, *23*, *40*). In the G protein recruitment assay, Ulotaront had a pEC_50_ value of 6.1, which is in agreement with the range of potencies (6.1 to 7.4) determined in previous studies (*22*, *40*–*42*). Compounds **30** and **65** were >25-fold more potent than Ulotaront and, hence, represented promising leads for further evaluation. In compound **65**, a methyl group was introduced onto the 4-position of the piperideine ring of compound **30**. We also evaluated **65** in a cyclic adenosine 3′,5′-monophosphate (cAMP) accumulation assay (fig. S11), and the compound showed a high potency (pEC_50_ = 9.4).

### Selectivity and ADME profile of TAAR1 agonists

Apart from the dopamine 2 (D_2_) receptor, antipsychotics tend to bind to several other aminergic GPCRs at therapeutically relevant concentrations. A multi-target profile can contribute to both favorable [e.g., enhanced antipsychotic efficacy and less motor disruption from additional 5-hydroxytryptamine 2A (5-HT_2A_) receptor blockade] and unfavorable (e.g., weight gain due to H_1_ receptor antagonism) effects of the drug (*43*). We evaluated the activity of compounds **30** and **65** at a panel of 36 aminergic GPCRs using a modified version of the PRESTO-TANGO assay (see Materials and Methods) (*44*). The assays were performed with positive controls (table S5) in each case, and we observed a satisfactory differentiation between the vehicle and control-treated samples (10 μM of reference agonist) for 27 receptors. The activities of compounds **30** and **65** and Ulotaront were then evaluated. Ulotaront partially activated 5-HT_1B_ (45% of control), 5-HT_1D_ (65%), 5-HT_1E_ (86%), α_2A_ (77%), and D_2_ (60%) receptors at 10 μM (Fig. 3D and figs. S5 to S8). These results are in agreement with previous experiments, in which Ulotaront activity at the 5-HT_1D_ and α_2A_ receptors was observed (*42*). Ulotaront has also been described as a 5-HT_1A_ agonist (*38*, *42*, *45*), but we did not observe a considerable activation of this receptor in the PRESTO-TANGO assay with Ulotaront or compounds **30** or **65** despite a considerable response to 5-HT. One reason for this result could be different optimal incubation times between the compounds and 5-HT. Compounds **30** and **65** exhibited a selectivity profile similar to Ulotaront with agonism at the 5-HT_1D_, α_2A_, and D_2_ receptors. Furthermore, compound **30** showed agonist activity at the H_1_ receptor (70%) and activated several members of the muscarinic acetylcholine receptor family, e.g., the M_2_ receptor (55%).

The panel screens of compounds **30** and **65** enabled us to prioritize receptors for further characterization of off-target activities. Potencies at four aminergic receptors including the D_2_ receptor were determined from concentration-response curves (Fig. 3E). Both compounds **30** and **65** were active at the D_2_ receptor (*E*_max_ of 54 and 59%, respectively). However, the observed potency of either compound was relatively weak (pEC_50_ values < 6 compared to the reference agonist quinpirole, pEC_50_ = 9.0). We also observed activity at the 5-HT_1D_ receptor (*E*_max_ of 52 and 75%, respectively; pEC_50_ < 6 for both; 5-HT, pEC_50_ = 7.1). Compound **65** was a full agonist of the α_2A_ receptor with modest potency (pEC_50_ = 6.0, *E*_max_ = 110%), and compound **30** was a low potency agonist of the M_2_ receptor (pEC_50_ = 5.0, *E*_max_ = 125%). None of the tested compounds showed notable antagonistic activity at the 5-HT_2A_ receptor compared to the reference antagonist ritanserin (fig. S9). None of the compounds exhibited agonism at the 5-HT_1B_ receptor, whereas only compound **65** lacked agonism at the H_1_ receptor (fig. S10).

On the basis of the high potency at TAAR1 combined with the lack of agonistic properties at undesirable targets such as the H_1_ and M_2_ receptors, in vitro absorption, distribution, metabolism, and excretion (ADME) properties of compound **65** were determined. Compound **65** showed excellent solubility (50 mM) and low plasma protein binding (fraction unbound of 61 and 81% in human and mouse, respectively). The molecule also had good permeability in a Caco-2 cell assay [Apparent permeability (*P*_app_) in the apical-to-basal (AB) and basal-to-apical (BA) directions: *P*_app_ AB = 7.7 × 10^−5^ cm/s and *P*_app_ BA = 16.8 × 10^−5^ cm/s] with slight efflux (efflux ratio = 2.2) and a favorable metabolic stability in the presence of human and mouse liver microsomes (intrinsic clearance CL_int_ = 16 and 7 μl/min per mg, respectively). We further characterized the pharmacokinetic profile of compound **65** in mice. Following intraperitoneal administration of 1 mg/kg, a maximum plasma concentration (*C*_max_) of 850 ± 172 nM was reached within 5 min (fig. S12, A and B). Plasma concentrations exhibited a monophasic decline up to 2 hours after administration, with a half-life of 17 min. By 4 hours, concentrations fell below the lower limit of quantification for both plasma (<5 nM) and brain (<40 pmol/g). Clearance of compound **65** after intraperitoneal administration was estimated at 225 ml/min per kg, with a corresponding large volume of distribution (*V*) of 6.3 liter/kg. Rapid distribution into the brain was observed, with the maximum concentration of 4707 ± 766 pmol/g reached within 5 min, mirroring the plasma concentration-time profile. The brain-to-plasma partition coefficient (total concentrations), *K*_p,brain_, was determined to be 7.9. These results indicated that compound **65** was suitable for further evaluation in vivo.

### In vivo antipsychotic-like activity of TAAR1 agonists

**Fig. 4.**
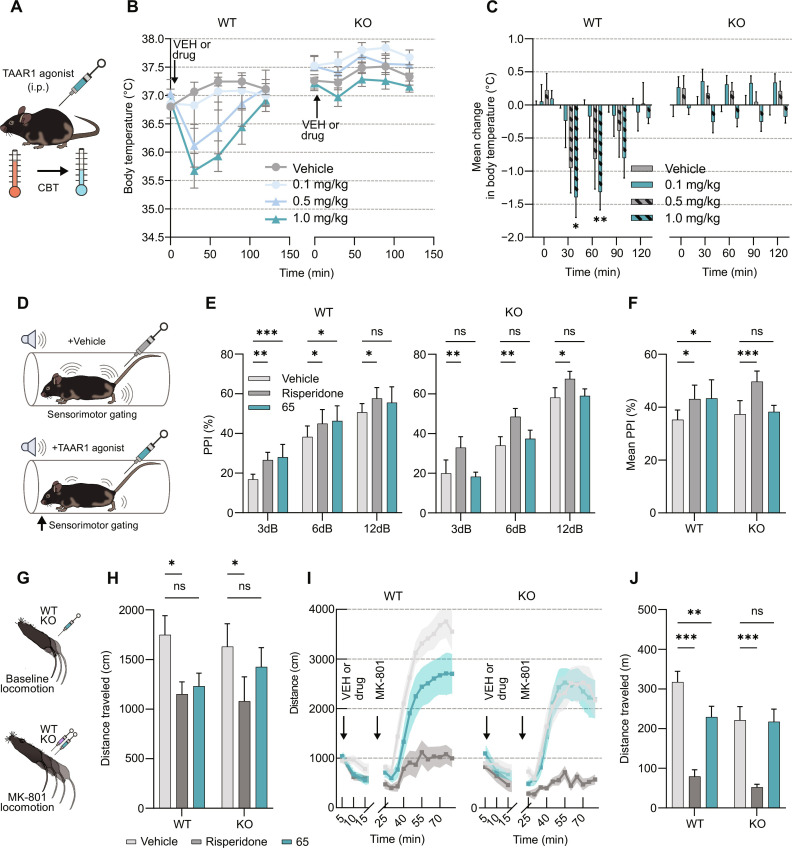
In vivo efficacy and antipsychotic-like activity of compound 65. (**A**) Evaluation of CBT change was performed in WT and TAAR1-KO mice after intraperitoneal injection of increasing doses of compound **65** (0.1 to 1 mg/kg, *n* = 6 to 7 mice per group). i.p., intraperitoneal. (**B**) CBT was recorded at 30-min intervals over a 120-min period following injection of different doses of compound **65** or vehicle (VEH) in both genotypes. (**C**) Average CBT shift [from (B)]. *P < 0.05 and **P < 0.005 (**65** versus vehicle) based on a two-way analysis of variance (ANOVA), Bonferroni’s multiple comparisons test. (**D**) PPI was examined in WT (*n* = 7) and TAAR1-KO (*n* = 8) mice after intraperitoneal injection of compound **65** (1 mg/kg), Risperidone (0.2 mg/kg), or vehicle. (**E**) PPI% at each pre-pulse in WT (left) or TAAR1-KO (right). *P < 0.05, **P < 0.005, and ***P < 0.001 (**65** or risperidone versus vehicle) based on a two-way ANOVA, Bonferroni’s multiple comparisons test. (**F**) Average change in PPI (%) across all pre-pulses in WT and TAAR1-KO mice. *P < 0.05 and ***P < 0.001 (**65** or risperidone versus vehicle) based on a two-way ANOVA, Bonferroni’s multiple comparisons test. (**G**) Locomotion experiments were performed in both WT (*n* = 10) and TAAR1-KO mice (*n* = 10). (**H**) Effect of compound **65** (1 mg/kg) or risperidone (0.03 mg/kg) on WT and TAAR1-KO baseline locomotion in the open-field test [*P < 0.05 (**65** or risperidone versus vehicle) based on a two-way ANOVA, Bonferroni’s multiple comparisons test] assessed for 10 min before MK-801 injections (0.35 mg/kg) as shown in (**I**) where time bins indicate 5-min intervals. ns, not significant. (**J**) Effects of compound **65** or vehicle on suppression of MK-801–induced hyperlocomotion. **P < 0.005 and ***P < 0.001 (**65** or risperidone versus vehicle) based on a two-way ANOVA, Bonferroni’s multiple comparisons test. See table S6 for more details on statistical analysis. All error bars represent means ± SEM.

To further assess the therapeutic potential of compound **65**, we evaluated the effect of this agonist in wild-type (TAAR1-WT) and TAAR1 knockout (TAAR1-KO) mice. We have previously reported a reduction of core-body temperature (CBT) mediated by TAAR1 activation as a tool to evaluate in vivo activity of TAAR1 agonists (*46*). Because of the high potency of compound **65** observed in our in vitro assays, we measured CBT in TAAR1-WT and KO mice at several doses (0.1, 0.5, and 1 mg/kg), which were lower than previously used with Ulotaront (10 mg/kg) (Fig. 4A) (*38*). In TAAR1-WT mice, compound **65** resulted in a reduction of CBT at doses of 0.5 and 1 mg/kg, reaching its peak 30 min after injection and returning to baseline after 2 hours. Conversely, no decrease in CBT was observed in TAAR1-KO mice (Fig. 4B). The extent of reduction (~1°C) was statistically significant at both 30- and 60-min intervals after injection in WT but not in TAAR1-KO mice using a dose of 1 mg/kg (Fig. 4C). We validated the responsiveness of TAAR1-KO mice to 8-hydroxy-2-(di-n-propylamino)tetralin (8-OH-DPAT), a known CBT reducing pharmacological agent targeting another receptor (5-HT_1A_ receptor, fig. S13A) (*47*, *48*). Last, we did not observe a significant increase in the maximal reduction of CBT elicited with an injection of 5 mg/kg versus 1 mg/kg (fig. S13B). Consequently, we used a dose of 1 mg/kg of compound **65** in the behavioral assays.

To assess whether compound **65** could exert antipsychotic-like effects, we conducted experiments in both WT and TAAR1-KO mice using the pre-pulse inhibition (PPI) behavioral assay. This aimed to ascertain whether the TAAR1 agonist, akin to Ulotaront (*38*), could enhance sensorimotor gating (Fig. 4D). In WT mice, treatment with compound **65** elevated PPI at both 3- and 6-dB pre-pulse levels (Fig. 4E) and showed similar effects to the atypical antipsychotic risperidone. Both compounds exhibited a general augmentation in PPI compared to the vehicle control (Fig. 4F) in WT mice. Consistent with our CBT data earlier, we did not observe any change in PPI at any specific or averaged pre-pulse intensity levels in TAAR1-KO mice following administration of compound **65**. In contrast, risperidone increased PPI also in TAAR1-KO mice.

Similarly, we assessed the locomotor effects of the compound because TAAR1 agonists have been shown to reduce baseline and psychostimulant induced hyperlocomotion (*49*, *50*). We measured locomotion both before (baseline) and after treatment with the *N*-methyl-d-aspartate receptor antagonist MK-801 in mice pretreated with vehicle, compound **65**, or risperidone. In WT mice, we noticed a trend toward decreased locomotion at baseline with compound **65** administration compared to the vehicle (Fig. 4H) and a notable reduction with risperidone. In addition, both compound **65** and risperidone led to a significant decrease in MK-801–induced hyperlocomotion in WT mice, although the inhibition observed in the compound **65**–treated group was less pronounced. Conversely, in TAAR1-KO mice, risperidone maintained a significant reduction in locomotion, whereas compound **65** did not exhibit such an effect (Fig. 4, H to J). To eliminate the potential of sedation influencing these observed results, WT and TAAR1-KO mice were treated with compound **65**, the sedative xylazine, or vehicle before the rotarod test. In both genotypes, compound **65** did not influence rotarod performance as opposed to xylazine (fig. S14). Collectively, our in vivo behavioral results suggest that compound **65** exerts antipsychotic-like effects and that these actions are TAAR1 specific.

### Comparison of models to the first cryo-EM structures of TAAR1

After this study was submitted for publication, several experimental structures of TAAR1 were released and revealed the binding site of agonists (*41*, *51*–*53*). The AlphaFold and homology models were aligned to the cryo-EM structures to assess the accuracy of our computational predictions. We calculated the average root mean square deviation (RMSD) from the experimental structures for the extracellular region and the orthosteric binding site, which are most relevant to virtual screening applications (table S7). AlphaFold outperformed homology modeling in the extracellular part of the receptor, including more accurate predictions of the TM region (Cα: 0.7 and 1.1 Å), loops (Cα: 2.8 and 4.1 Å), and binding site (Cα: 0.7 and 0.9 Å; side chains: 1.4 and 1.6 Å). It should be noted that the experimental TAAR1 structures were determined in complex with a G protein, and, as AlphaFold predicted an overall inactive receptor state, the homology models were closer to experiment in the intracellular region due to the selection of an template in an active conformation. The considerably higher structural accuracy of the AlphaFold predictions in the extracellular region was consistent with the improved hit rate from virtual screens using these models.

The experimental TAAR1 structures revealed the binding modes of the endogenous ligand β-PEA and synthetic agonists. β-PEA was anchored by a salt bridge with D103^3.32^, and the phenyl ring was located in the pocket created by V184^45.52^, F186^45.54^, T194^5.42^, S198^5.46^, F267^6.51^, and F268^6.52^. The 10 synthetic agonists established similar interactions in the orthosteric site, and eight of these were based on the β-PEA scaffold. The most potent agonists from the AlphaFold docking screen (**27**, **29**, and **30**) were predicted to occupy the same pocket (Fig. 2), but none of these compounds contained the phenethylamine core. Instead, the three docking hits were derived by combining a smaller aromatic ring (furan and thiophene) with a six-membered ring bearing a positive charge (morpholino and 3-piperideine), which are characteristic features of aminergic GPCR ligands (*32*). Morpholino and thiophene groups were also present in Ralmitaront [Protein Data Bank (PDB) accession code: 8JLP] and Ulotaront (PDB accession code: 8W88), respectively (*41*, *52*). These groups occupied the same subpockets in both the experimental structures of these agonists and computational models of compounds **27** and **29**.

**Fig. 5.**
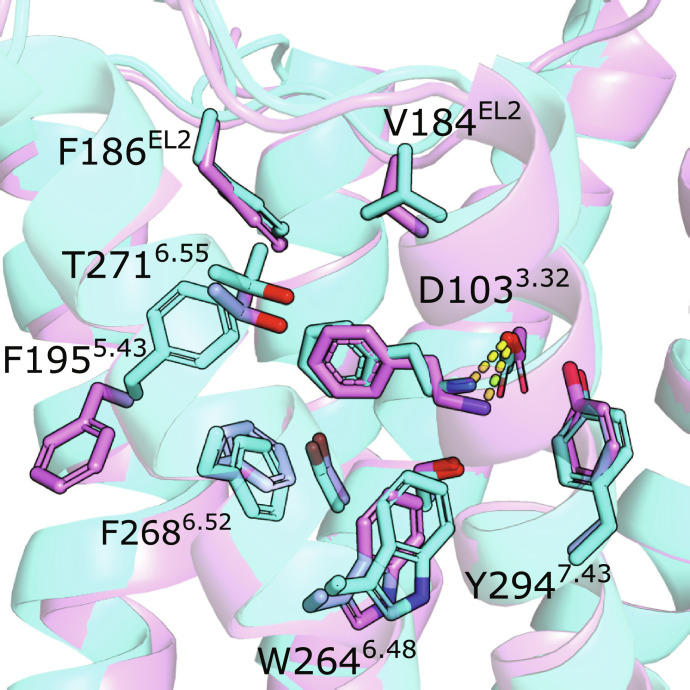
Predicted and experimental structure of TAAR1 in complex with β-PEA. Comparison of an AlphaFold model of TAAR1 (cyan) to the cryo-EM structure with PDB accession code 8W89 (violet). The structures have been aligned based on the binding site residues. The receptor is shown as a cartoon. Binding site side chains and the ligand are shown as sticks. Polar interactions are shown as yellow dashed lines.

The AlphaFold models used in the docking screen were most similar to the structures of TAAR1 in complex with smaller agonists such as β-PEA (PDB accession code: 8W89). In these models, the binding site residues adopted similar rotamers as in the experimental structures, with one exception (Fig. 5). The side chain of F195^5.43^ consistently adopted different conformations, which led to a slightly larger orthosteric site in the experimental structures. However, none of the ligands extended into this relatively narrow pocket, and the F195^5.43^ conformation did not appear influence the docking pose of β-PEA, which was predicted with high accuracy by docking to the AlphaFold models (average RMSD = 1.0 Å, Fig. 5). We also docked compound **30** to the cryo-EM structure and identified a low-energy binding mode similar to that obtained using the AlphaFold model (fig. S15). Docking of the set of ligands and decoys using the cryo-EM structure of TAAR1 in complex with β-PEA resulted in high enrichment (LogAUC = 24 and EF1% = 13). The experimental structure hence performed better than the median enrichment of the AlphaFold and homology models (LogAUC = 19 and 15, respectively, Fig. 1B). The AlphaFold models that were used in the prospective screen performed slightly better than the experimental structure in this assessment (LogAUC = 27 to 32 and EF1% = 17 to 22).

The larger synthetic ligands altered the size and shape of the TAAR1 binding site. Several of these compounds extended into cavities that were not present in the complex with β-PEA. For example, Ralmitaront occupied a pocket in the interface between TM4 and TM5, and the bulky adamantine group of A77636 altered the conformation of EL2. Notably, these conformations were not captured by any of the 1000 generated AlphaFold models of TAAR1. Because of the smaller size of the pocket in the AlphaFold models and experimental structure of TAAR1 bound to β-PEA, several of the larger synthetic agonists (e.g., Ralmitaront, RO5256390, and A77636) could not be docked into these structures because of steric clashes.

## DISCUSSION

Three main results emerged from our virtual screens based on computational models of TAAR1, a promising therapeutic target for central nervous system disorders. First, the ligand binding sites of the homology and AlphaFold models had different sizes and shapes, which strongly influenced the results of structure-based virtual screening. By analyzing thousands of predicted TAAR1 structures, we identified the most suitable models for virtual screening. Second, prospective docking screens using the two types of models identified diverse TAAR1 agonists in large chemical libraries. Notably, the receptor structures predicted by AlphaFold resulted in a high hit rate of 60%, which was more than twofold higher than that achieved by the homology model. Last, one of the most potent agonists showed a promising selectivity profile in cellular studies and exhibited antipsychotic-like effects in several rodent models.

The structural accuracy of AlphaFold is comparable to experimental methods, but several studies have questioned whether it is genuinely useful in ligand design (*11*, *12*, *14*, *15*). Whereas previous benchmarks of virtual screening performance have compared the predictions of AlphaFold to experimental structures (*14*, *15*), the focus of our study was to determine whether AlphaFold presents advantages over homology modeling, which has been the state-of-the-art method for GPCR structure prediction (*25*, *54*). In addition, our approach was also unique in that screens for ligands were performed, allowing a direct comparison of the two types of models. Encouragingly, the hit rates of the docking screens for the homology and AlphaFold models were high (22 and 60%, respectively), and potent TAAR1 agonists were discovered in each case. This result reflects the fact that the aminergic GPCR family, including TAAR1, has buried binding pockets that are ideal for structure-based ligand discovery (*6*). Our data can also be compared to docking screens performed using crystal and cryo-EM structures of other aminergic GPCRs, which yielded high hit rates for adrenergic (53 and 63%) (*8*, *55*), dopamine (56%) (*56*), and histamine (73%) (*57*) receptors. Moreover, Lyu *et al.* (*58*) recently compared prospective docking screens using cryo-EM structures and AlphaFold models of the 5-HT_2A_ receptor, resulting in comparable hit rates of 23 and 26%, respectively. Hence, AlphaFold structures can outperform homology models in virtual screening applications and offer results comparable to experimental GPCR structures.

A limitation of AlphaFold is that the algorithm was designed to predict a single static structure of a protein. This overlooks the dynamic nature of proteins and the multiple functional states of GPCRs (*59*), which can influence the performance of molecular docking. Previous studies have indicated that the poor performance of AlphaFold models in docking calculations is due to small variations in the shape of the binding site, which is mainly due to side-chain conformations (*11*, *13*, *14*). The TAAR1 AlphaFold models, on average, performed worse than one of recently released experimental structure of the receptor in retrospective ligand enrichment calculations (*41*). This result agrees with previous studies comparing AlphaFold models to experimental structures, which concluded that further refinement of the binding sites is needed to improve virtual screening performance, e.g., using induced-fit docking of known ligands (*14*). One of our main results is that binding site structures suitable for virtual screening can also be identified by evaluating a large number of AlphaFold models. In addition, the risk of selecting a poorly performing AlphaFold model can be further reduced by using an ensemble of binding site structures in the prospective virtual screen. Encouragingly, one of the TAAR1 cryo-EM structures released after our virtual screens closely resembled our AlphaFold models. However, the cryo-EM structures also revealed that diverse shapes of the binding pocket were stabilized by the largest synthetic ligands, which would have been valuable information in the optimization of the docking hits (*41*, *52*). The alternative binding site conformations were not predicted by AlphaFold. These results suggest that our approach could be further improved by using methods that generate more diverse ensembles of structures or fold the receptor in the presence of small molecules (*60*, *61*). The models of the TAAR1 binding site were selected on the basis of the enrichment of known agonists, which appeared to bias the outcome of the prospective screen. All the discovered TAAR1 ligands were agonists, indicating that the models represented an active binding site structure. In this context, it should be noted that the TAAR1 structure predicted by AlphaFold represented an overall inactive receptor conformation. Recently developed AlphaFold-based methods that enable prediction of either inactive or active GPCR conformations could further improve model accuracy (*62*, *63*).

We selected TAAR1 as the target of our study because the (unknown) structure was likely to be accurately predicted by both homology modeling and AlphaFold, enabling a fair comparison of virtual screening performance. However, if no suitable templates are available, then AlphaFold can be expected to be substantially more accurate than homology modeling and also requires considerably less effort (*1*, *2*, *11*, *64*). Atomic resolution structures are lacking for 52% of the non-sensory GPCRs, corresponding to 188 unique receptors, and the approach used in this work can be applied to the 86 targets for which there is at least one known ligand. Our retrospective docking calculations on thousands of TAAR1 models also show that ligand enrichment could be expected even if the model selection was not guided by known actives, indicating that virtual screening for ligands of orphan receptors may also be enabled by AlphaFold. In the case of TAAR1, the AlphaFold models outperformed homology modeling in both the retrospective and prospective docking screens. Benchmarking on a larger set of diverse GPCRs would be required to assess if this finding is generally true. At this point, we would encourage the exploration of both AlphaFold and homology models because the optimal choice of method may be target dependent. However, together, our observations clearly indicate that the highly accurate AlphaFold models should provide unprecedented opportunities to discover ligands of drug targets using virtual screening.

TAAR1 has emerged as a promising target for the treatment of neuropsychiatric disorders, and the pharmaceutical industry initially focused on schizophrenia (*17*, *18*). Although Ulotaront did not meet the primary endpoints in the treatment of acutely psychotic adults with schizophrenia in two recently completed phase 3 trials (possibly due to a high placebo response), this compound continues to be advanced in clinical investigation, notably for Parkinson’s disease psychosis, narcolepsy, generalized anxiety disorder, and major depressive disorder (*18*, *21*, *46*, *49*). The fact that some TAAR1 agonists, including compound **65**, appear to exert antipsychotic-like properties without potent antagonism at the D_2_ dopamine receptor makes them particularly suitable for the treatment of patients with Parkinson’s disease psychosis or Lewy body dementia because such patients also require pro-dopaminergic therapies to control their parkinsonism. Considering that some of our TAAR1 agonists, as well as previously identified compounds (*65*, *66*), contain privileged structures for antipsychotic activity, it also appears possible that other investigational and approved psychotropic drugs could modulate TAAR1 activity. This warrants further investigation, and Zilberg *et al.* (*51*) recently discovered that the antipsychotic asenapine is a potent TAAR1 agonist.

The key result from our study is that molecular docking screening identified GPCR ligands by using machine learning–predicted structures, which could accelerate drug discovery for novel targets such as TAAR1. One of the discovered TAAR1 agonists, compound **65**, is more potent at TAAR1 than Ulotaront and shows promising selectivity and good pharmacokinetic properties. We also compared the selectivity of compound **65** to Ulotaront, and these agonists exhibited a similar profile in a panel of aminergic GPCRs. The in vivo data show that compound **65**, similarly to other TAAR1 agonists, lowers the CBT, increases sensorimotor gating in the PPI behavioral assay, and inhibits MK-801–stimulated hyperlocomotion. Notably, these effects were observed at a lower dose (1 mg/kg) than in previous studies of Ulotaront (3 to 30 mg/kg) (*42*) and were absent in TAAR1-KO mice, which suggests that this lead exerts antipsychotic-like effects by modulating TAAR1 activity. Hence, this scaffold represents a promising therapeutic lead for the development of medications for the improved management of neuropsychiatric disorders.

## MATERIALS AND METHODS

### Computational methods

#### Homology and AlphaFold models

Sequence alignments and homology models were generated using MODELLER (version 10.2) (*67*) and the human TAAR1 sequence from UniProt (Q96RJ0) (*68*). The TAAR1 sequence was first aligned to each of the four selected templates (table S1). Manual adjustments to the alignment were then made in EL2 based on an analysis of available experimental structures of aminergic GPCRs. The third intracellular loop, N terminus, and C terminus were excluded because these regions could not be predicted on the basis of the template structures. A set of 1000 TAAR1 homology models based on four templates was first generated (250 models/template, table S1). The homology modeling protocol for the β_1_ adrenergic receptor template [PDB code: 7BU6; (*69*)] was optimized by generating 1000 structures and introducing a dihedral restraint on the side chain of F186^45.54^ (χ_1_ angle C-Cα-Cβ-Cγ, 5 kcal/mol, phase of 315°, and period of 3) based on docking to representative models and analysis of available experimental GPCR structures, as described previously (*26*, *27*). The homology models were prepared for molecular docking by adding polar hydrogens and assigning histidine protonation states using PrepWizard (*70*). The 1000 AlphaFold models were generated with AlphaFold2 (version 2.1, default settings) (*1*) using the full TAAR1 sequence, and polar hydrogens and histidine protonation states were maintained in the docking calculations. After the release of the TAAR1 cryo-EM structures (PDB accession codes: 8JLN, 8JLO, 8JLP, 8JLQ, 8JLR, 8JSO, 8UHB, 8W87, 8W88, 8W89, 8W8A, 8WC8, and 8WCA) (*41*, *51*–*53*), alignments of the homology and AlphaFold models to the experimental coordinates and symmetry-corrected calculations of RMSD values were performed using the PyMol version 2.5, in-house scripts, and spyrmsd (*71*).

#### Molecular docking screens

Molecular docking calculations were performed using DOCK3.7 (*24*, *37*). The flexible-ligand docking algorithm used by DOCK3.7 matches rigid segments of a molecule’s conformational ensemble onto a set of receptor matching spheres, which define the binding site. The matching spheres were generated on the basis of the predicted binding modes of known agonists, which were obtained by docking to a representative TAAR1 model using Glide SP (*72*). Between 26 and 45 spheres were obtained for the final 2000 TAAR1 models (1000 structures for each type of model). A physics-based scoring function was used to predict the binding energy of each docked compound, which was estimated as the sum of protein-ligand interaction energies (electrostatic and van der Waals) and a ligand-desolvation term (*31*). The partial charges and van der Waals parameters were derived using an AMBER united-atom force field (*73*). Pre-generated scoring grids were generated using QNIFFT (electrostatic interaction energies) (*74*), CHEMGRID (van der Waals interaction energies) (*75*), and SOLVMAP (ligand desolvation energies) (*31*). The solute-solvent dielectric interface was extended by a 0.6-Å radius from the binding site surface in the calculations of electrostatic interaction energies and desolvation penalties (*37*). In-house scripts were used to generate property-matched decoys (*76*) to a collection of TAAR1 ligands obtained from the ChEMBL database (source description: Scientific literature, potency < 10 μM, molecular weight < 250 Da) and scientific articles (*22*, *29*). The 173 ligands and 11,392 decoys were prepared for docking with DOCK3.7 using the ZINC database protocol (*35*). The models generated with each method and a TAAR1 cryo-EM structure (PDB accession code: 8W89) were evaluated by assessing ligand enrichment, which was quantified by calculating LogAUC and EF1% from receiver-operator characteristic curves. Five models per method were selected from the set of best enriching models (98th percentile) and used in the prospective virtual screens.

In each virtual screen, a library of 16 million fragment-like compounds from the ZINC database (Log*P* <3.5 and molecular weight < 250 Da) (*35*) was docked to the orthosteric site of the five TAAR1 models. Each docked compound was fit into the active site in an average of ~14,000 orientations, and, for each orientation, an average of 208 conformations were sampled. The top-scoring pose of each compound was further refined using a simplex rigid-body minimization. Each molecule was assigned the best docking score among the five models, followed by ranking of the library compounds. The 40,000 top-ranked compounds from each screen were then filtered in several steps. Substructures present in PAINS (*36*) and compounds with *T*_c_ value (ECFP4-based fingerprints) greater than 0.5 to known TAAR1 agonists were excluded. Last, the remaining compounds were clustered using ECFP4-based fingerprints (Morgan, 1024 bits, radius = 2) and a *T*_c_ value of 0.5 (*37*), leading to 11,779 and 8995 clusters for the AlphaFold and homology model screen, respectively. Of these, the 2000 top-ranking clusters were inspected visually to select compounds for experimental evaluation.

#### Structural coverage of the GPCR family and availability of ligands

GPCRs of unknown structure with at least one known drug-like ligand were identified using three sets of data from GPCRdb (*54*). The list of all human GPCRs was first retrieved using GPCRdb (https://gpcrdb.org), and, then, all sensory and olfactory receptors were excluded. Ligands of the remaining receptors with a reported activity ≤ 10 μM [inhibition constant (*K*_i_), dissociation constant (*K*_d_), median inhibitory concentration (IC_50_), or median effective concentration (EC_50_) values] and a molecular weight ≤ 500 Da were collected from GPCRdb. Last, all GPCR structures determined by x-ray crystallography or cryo-EM methods were obtained from GPCRdb.

### Experimental methods

#### In vitro studies

##### Cell culture

Expi293F cells (provided by I. Kotliar, Rockefeller University) were maintained in 8% CO_2_, 37°C, and 95% humidity and 125-ml shaking flasks (Corning) using Gibco Expi293 Expression Medium. HTLA cells, a HEK293-derived cell line containing stable integrations of a tTA-dependent luciferase reporter and a beta-arrestin2 TEV fusion gene, (shared by B. Roth, University of North Carolina) were maintained in T175 flasks (Sarstedt) with Dulbecco’s modified Eagle medium (DMEM) supplemented with 10% fetal bovine serum (FBS), GlutaMAX, sodium pyruvate, Hepes, nonessential amino acids, and penicillin-streptomycin (Gibco).

##### G protein recruitment assay

To monitor TAAR1 activation by compounds, we used our previously described system. Briefly, 48 hours after transfection with LgBiT tagged mini-Gα_s_-393 and C-terminally SmBiT-tagged hTAAR1, Expi293F cells were diluted to a total volume of 10 ml in Dulbecco’s phosphate-buffered saline (DPBS) and 10 μM of coelenterazine-h (NanoLight). Cells were then reseeded in solid white, 96-well plates (Corning) with 90 μl of cell suspension per well. Luminescence was read using a plate reader for 5 min (SPARK 10 M, Tecan) before the addition of compounds to ensure signal stability. Ten microliters of each compound diluted in DPBS was added, and luminescence from each well was read for 10 min, with 100-ms read time per well. The A_2A_R counter-screen was performed using the same protocol. In these experiments, the hTAAR1 was replaced by a C-terminally SmBiT-tagged hA_2A_R construct.

##### Aminergic receptor screen

Assessment of receptor activation by compounds at all human aminergic receptors were carried out using a modified version of the PRESTO-TANGO method. Briefly, 5 μl of each TANGO-ized GPCR plasmid (sourced via Addgene, kit no. 1000000068) at a concentration of 10 ng/μl was spotted per well (CulturPlate-384, White Opaque, PerkinElmer) using a liquid handling robot (Apricot S3, SPT Labtech). The plate was spun down, and 5 μl of a 1:25 dilution (in DPBS) of polyethylenimine (PEI) derivative (Transporter 5, Polysciences) was added (1:4 cDNA to PEI ratio) and incubated for 20 min at room temperature (RT). HTLA cells (human embryonic kidney 293 cell line that expresses tetracycline transactivator–dependent luciferase reporter) were washed twice with pre-warmed DPBS and then resuspended to 562 cells/μl in FluoroBriteDMEM and 5% dialyzed FBS (One Shot format, Gibco) and 1% penicillin-streptomycin (10,000 U/ml, Gibco). The plate was spun down, and 40 μl of the resuspended cells was added to each well with an electronic multichannel pipette (E1-ClipTip, Thermo Fisher Scientific) and incubated for 24 hours at 37°C and 5% CO_2_ in a humidity chamber. After 24 hours, 5 μl of compound (11× stock) was added with an electronic multichannel pipette and incubated for 16 hours at 37°C and 5% CO_2_ in a humidity chamber. After 16 hours of incubation, 6 μl (1:10 dilution) of BrightGlo (Promega) was added. Luminescence signal was detected using a plate-reader (SPARK, Tecan) with 100-ms integration time and a 100-ms delay between well reads. For follow-up concentration-response curve measurements, HTLA cells were transfected in suspension with medium at a concentration of 1 μg of cDNA/ml and 562 cells/μl. Fifty microliters of the suspension was added per well to white 384 well plates and incubated for 24 hours before stimulation for 16 hours with compounds. For antagonist mode, cells were pretreated for 15 min with the compounds before the addition of an agonist concentration corresponding to its approximate EC_80_ at that receptor (experimentally determined beforehand).

##### cAMP accumulation assay

cAMP accumulation was assessed using a previously described system with some modifications (*45*). hTAAR1 and GloSensor-22F were transected with a 1:1 gene dose (1 μg/ml) in Expi293F cells for 48 hours. Cells were then resuspended in assay buffer [Hanks’ balanced salt solution (HBSS), 10 mM Hepes, 2 mM d-Luciferase (Promega), and 300 μM 3-isobutyl-1-methylxanthine (Sigma-Aldrich)] and seeded with 25,000 cells per well to a white 384-well plate (CulturPlate, PerkinElmer) and incubated for 1 hour in the dark at RT. After a baseline read, compounds were added, and the plate was read for a further 1 hour (SPARK 10 M, Tecan) with 10-min intervals. The final read at 1 hour was used for analysis.

#### In vivo studies

##### Animals and housing conditions

TAAR1-KO animals were generated as previously described (*49*). Male and female (3 to 4 months old for PPI experiments and 3 to 6 months old for other experiments) WT and TAAR1-KO mice were used for testing. Naïve cohorts of animals were used for the different behavioral/physiological tests. For open-field, CBT, and PPI tests, separate cohorts were used, while repeated measures in the same mice were used for the different treatment or dose conditions. The mice were accommodated in rooms maintained at a constant temperature (20°C) and humidity (53%), adhering to a 12-hour light/dark cycle. They were provided with access to food pellets and water ad libitum. Tests were performed during the light phase of the light/dark cycle. All experiments were approved by the local ethical committee at Karolinska Institute (N3218-2022) and conducted in accordance with the European Communities Council Directive of 24 November 1986 (86/609/EEC).

##### Preparation and dosing of compounds

Compound **65** was dissolved in 5% dimethyl sulfoxide (DMSO) in saline and administered at a dose of 0.1 to 5 mg/kg as indicated in each experiment. Risperidone (R3030, Sigma-Aldrich) was dissolved in 5% DMSO in saline and administered at a dose of 0.2 mg/kg for PPI tests and 0.03 mg/kg for open-field tests (OFTs). MK-801 hydrogen maleate (M107, Sigma-Aldrich) was dissolved in saline and administered at a dose of 0.35 mg/kg. Xylazine (Rompun, Elanco Denmark) was diluted in saline and administered at a dose of 5 mg/kg. Injection volumes were 10 ml/kg given intraperitoneally.

##### In vivo pharmacokinetics

Male C57BL/6 mice were administered compound **65** intraperitoneally at 1 mg/kg. Mice were sacrificed at 5, 30, 60, 120, 360 and 1440 min after dosing (*n* = 3 per time point). Brains were collected and snap-frozen. Blood was sampled from the cervical vein upon decapitation in Eppendorf tubes containing 50 μl of 0.5 M EDTA and immediately centrifuged for 10 min at 3000*g* and 4°C. Isolated plasma was then transferred into clean polypropylene tubes. All samples were stored at −20°C before analysis.

The concentration of compound **65** in plasma samples and brain tissue was determined by the liquid chromatography/tandem mass spectrometry (LC/MS-MS) technique (Waters Corp., MA, USA) after protein precipitation. Brains were weighed and homogenized 1:3 (w/v) in PBS using a Minilys Bead Homogenizer (Bertin Instruments). Analytical calibration standards and quality control samples were prepared in blank plasma and brain homogenate by addition of compound **65** in the range of 5 to 2000 nM for plasma and 10 to 2000 pmol/gram tissue for brain, respectively. Samples were precipitated with acetonitrile (1:4) containing warfarin as analytical internal standard. The supernatants were injected into the LC-MS/MS system consisting of a TQ-S micro mass spectrometer coupled to an Acquity UPLC (Waters Corp, MA, USA). Analytical separation was performed using mobile phases (A) 0.1% formic acid and (B) 99.9% acetonitrile with 0.1% formic acid and an Acquity HSS T3 column (2.1 mm by 50 mm, 1.7 μm, Waters Corp., MA, USA). The obtained concentrations of brain homogenate samples were multiplied by 4 to account for the dilution during preparation of the homogenate. Pharmacokinetic parameters were determined from the experimental data by applying non-compartmental analysis using Microsoft Excel spreadsheet. The brain-to-plasma partition coefficient (*K*_p,brain_) was calculated as the ratio of the area under the concentration–time curves (AUC) in brain versus plasma (AUC_brain_/AUC_plasma_).

##### Body temperature measurement

Core temperature was determined by a rectal thermoprobe. Compounds or vehicle was administered intraperitoneally. A basal measurement was performed immediately before injection. Measurements were repeated every 30 min for 2 hours. Mean changes in CBT were calculated by subtracting the value from vehicle treated group at the corresponding time point. A washout of 1 week between different doses of compound **65** was applied, and mice were injected starting with the lowest dose first.

##### Pre-pulse inhibition

Measurements were performed using startle response chambers (San Diego Instruments, San Diego, CA, USA). Each chamber is sound-isolated and contains a Plexiglass animal enclosure attached to a platform and a loudspeaker that can produce both continuous white noise and acoustic pulses of varying intensities. The animal enclosure is large enough for the animal to turn around and make other movements to reduce restraint stress. Mouse startle responses to acoustic stimuli are transformed by a piezoelectric transducer under the platform into an analog signal. The transducers were calibrated before each experiment using a standardization unit (San Diego Instruments, San Diego, CA, USA), and the speakers were calibrated using a Type 2670 Microphone Preamplifier, a Type 4230 Sound Level Calibrator, and a Type 2610 Measuring Amplifier (Brüel & Kjær, Nærum, Denmark). To habituate the mice to the chambers, they were placed in the enclosure for 5 min, with 65-dB background white noise, on three different days before the start of experiments. PPI measurements were then conducted on each mouse on four occasions separated by 96 hours. The first occasion was a mock test session where all mice were injected with vehicle, serving to reduce the effect of novelty on subsequent sessions. The next three sessions were true test sessions with injection of either compound **65**, risperidone, or vehicle. The R package agricolae was used to generate a randomized block design, randomizing treatment order across sex, genotype, and test session.

Animals were brought to the testing room for habituation 60 min before the starting of tests. Thirty minutes before PPI measurements, mice were injected with either compound **65** (1 mg/kg), risperidone (0.2 mg/kg), or vehicle intraperitoneally. Each test session started with a 5-min background noise (65-dB white noise) habituation period. The background noise continued throughout the test session. The habituation period was followed by four blocks of trials, with the first and last blocks consisting of five pulse-only trials (40-ms, 120-dB pulse). During block 2, five trial types were presented five times each in a pseudo-randomized order. The trial types included pulse only, no stimulus (NOSTIM), and three different pre-pulse trials in which 20 ms pre-pulses of different intensities (68, 71, or 77 dB) preceded the startle pulse (120 dB) by a 100-ms interval. Block 3 contained the same numbers and types of trials as block 2, but in a different pseudorandom order. A variable intertrial interval averaging 12 s was used. The test session lasted for a total of 23 min and contained 60 trials in total. Animal enclosures were cleaned with 70% ethanol between each animal. PPI data are shown as PPI%, which was calculated as [1 − (pre-pulse trials/startle-only trials)] × 100. Mean PPI was calculated by adding the average PPI% for all pre-pulse intensities (3, 6, and 12 dB) and dividing it by the number (i.e., 3) of pre-pulse intensities.

##### Open-field test

To assess the influence of the compound on baseline locomotion and MK-801–induced hyperlocomotion, mice were subjected to open-field testing on four occasions separated by 96 hours. The first occasion was a mock test session where all mice were injected with vehicle only, serving to reduce the effect of novelty on subsequent sessions. The next three sessions were true test sessions with pretreatment of either compound **65**, risperidone, or vehicle, followed by MK-801. The R package agricolae was used to generate a randomized block design, randomizing treatment order across sex, genotype, and test session. After injection with either compound **65** (1 mg/kg), risperidone (0.03 mg/kg), or vehicle intraperitoneally, mice were immediately placed in the open field and recorded for 15 min. They were then injected with MK-801 (0.35 mg/kg intraperitoneal) and recorded again for 60 min. The OFT arena measured 46 cm by 46 cm with gray floor and walls. The brightness at the center of each arena was 30 to 50 lux. The arenas were cleaned with 70% ethanol between each animal. Video from a camera mounted in the ceiling was analyzed using EthoVision XT11.5 (Noldus) software.

##### Rotarod test

To test compound **65** for sedative effects, mice were subjected to the accelerating rotarod test as previously described (*77*), with minor modifications. Mice were acclimatized to the rotarod (Ugo Basile, Gemonio, Italy) for 2 days before testing, starting at 4 rotations per minute (RPM) and accelerating to 40 RPM over 300 s. A baseline measure of performance, defined as the latency to fall from the rotarod with a cutoff time of 300 s, was taken before injection with either compound **65** (1 mg/kg), xylazine (5 mg/kg), or vehicle intraperitoneally. The mice were then retested 30 min after injection. Mice that did not complete 100 s on the rotarod at baseline were excluded from the experiment.

#### In vitro ADME assays

##### Thermodynamic solubility

Thermodynamic solubility assay used solid form of compound **65**. Solid test compound (~2 to 3 mg) was weighed in a glass high-performance liquid chromatography (HPLC) vial, and 100 mM KPO_4_-buffer (pH 7.4) was added to give a theoretical max concentration if everything is dissolved of ~5 to 6 mg/ml. The vial was incubated at 900 rpm and 37°C in a rotational shaker for 24 hours. After the incubation, an aliquot (200 μl) was transferred to a glass insert and centrifuged at 10,000*g* and 37°C for 20 min to separate any solid material from the solution. The supernatant was transferred to a new HPLC vial and analyzed by LC-MS/MS.

##### Caco-2 cells permeability assay

Caco-2 cell monolayers (passages: 94 to 105) were grown on permeable filter support and used for transport study on day 21 after seeding. Before the experiment, a drug solution of 10 μM was prepared and warmed to 37°C. The Caco-2 filters were washed with pre-warmed HBSS before the experiment, and, thereafter, the experiment was started by applying the donor solution on the apical or basolateral side. The transport experiments were carried out at pH 7.4 in both the apical and basolateral chamber. The experiments were performed at 37°C and with a stirring rate of 500 rpm. The receiver compartment was sampled at 15, 30, and 60 min, and, at 60 min, also a final sample from the donor chamber was taken to calculate the mass balance of the compound. The samples (100 μl) were transferred to a 96-well plate containing 100 μl of methanol, and Warfarin as internal standard and was sealed until LC-MS/MS analysis.

##### Plasma protein binding and plasma stability in human and animal plasma

Pooled human plasma was provided by Uppsala Academic Hospital and was collected from two donors (nonsmoking) (citric acid). In brief, 0.2 ml of the plasma (50% plasma and 50% isotonic buffer) test solution (typically, 10 μM final compound concentration) was transferred to the membrane tube in the rapid equilibrium dialysis (RED) insert (Thermo Fisher Scientific). Isotonic phosphate buffer (0.35 ml; pH 7.4) was added to the other side of the membrane. The 96-well base plate was then sealed with an adhesive plastic film (Scotch Pad) to prevent evaporation. The sample was incubated with rapid rotation (>900 rpm) on a Kisker rotational incubator at 37°C for 4 hours to achieve equilibrium. A stability test of the test solution was prepared (to allow detection of drug degradation), and 100 μl of the plasma test solution (in a plastic vial or on a sealed plate) was incubated at 37°C for 4 hours (or as long as the dialysis time). The plasma test solution was frozen directly after the administration to prevent any degradation. Before LC-MS/MS analysis, the plasma and buffer samples were treated with the addition of methanol (1:3) containing Warfarin as internal standard to precipitate proteins. The standard curve was created using the plasma standard. The plate was then sealed and centrifuged, and the supernatant was analyzed by LC-MS/MS (*78*).

##### Metabolic stability in the presence of human and animal liver microsomes

Metabolic stability was determined in human (0.5 mg/ml; mixed gender pooled, XenTech LLC, KS, USA) or mouse (pooled CD1 male, XenoTech LLC, KS, USA) liver microsomes at a compound concentration of 1 μM in 100 mM KPO_4_ buffer (pH 7.4) in a total incubation volume of 500 μl. The reaction was initiated by addition of 1 mM reduced form of nicotinamide adenine dinucleotide phosphate. At various incubation times, i.e., at 0, 5, 10, 20, 40, and 60 min, a sample was withdrawn from the incubation, and the reaction was terminated by addition of cold acetonitrile with warfarin as an internal standard. The amount of parent compound remaining was analyzed by LC-MS/MS.

## Supplementary Materials

### This PDF file includes:

Experimental methods: Chemistry Figs. S1 to S23 Tables S1 to S7
