# Supplementary material for: AlphaFold accelerated discovery of psychotropic agonists targeting the trace amine–associated receptor 1
Source: Sci Adv. 2024 Aug 7;10(32):eadn1524. doi: 10.1126/sciadv.adn1524 (PMC11305387; doi:10.1126/sciadv.adn1524)
Supplement: Supplementary file 1 — Experimental methods: Chemistry Figs. S1 to S23 Tables S1 to S7 [file sciadv.adn1524_sm.pdf]

Science Advances
AAAS

Supplementary Materials for

AlphaFold accelerated discovery of psychotropic agonists targeting the trace amine–associated receptor 1

Alejandro Díaz-Holguín *et al.*

Corresponding author: Jens Carlsson, jens.carlsson@icm.uu.se; Per Svenningsson, per.svenningsson@ki.se

Sci. Adv. **10**, eadn1524 (2024)
DOI: 10.1126/sciadv.adn1524

This PDF file includes:

Experimental methods: Chemistry
Figs. S1 to S23
Tables S1 to S7

Experimental methods: Chemistry

Compound synthesis

Compounds **1-67** and **69-77** were synthesized by Enamine, and the purity of these were at least 90% based on LC/MS data. Compounds **30**, **68**, and **78** were synthesized in-house, as described in the following section. For the most potent compounds that were most extensively characterized in biological assays (**30** and **65**), purity was higher (≥95%), which was confirmed in-house by LC/MS and NMR spectroscopy.

General synthetic procedures

All reagents were purchased from Fluorochem, Sigma-Aldrich, Enamine and Chemtronica. DCM, methanol, DMF, and acetonitrile (99.9%) were purchased from VWR International AB, whereas THF was purchased from Sigma-Aldrich. Reagents and solvents were used as such without further purification. All reactions involving air or moisture-sensitive reagents or intermediates were performed under a nitrogen atmosphere. Mainly LC/MS was used for monitoring reactions using an Agilent 1100 series HPLC having a C18 Atlantis T3 column (3.0 × 50 mm, 5 μm). Acetonitrile–water (flow rate 0.75 mL/min over 6 min) was used as mobile phase and a Waters micromass Z_Q (model code: MM1) mass spectrometer with electrospray ionization mode was used for detection of molecular ions. TLC (Merck, silica gel 60 F_{254} plates) was sometimes used for monitoring reactions, particularly in the purification of compounds. Visualization of the developed TLC was done using UV light (254 nm) and staining with ninhydrin or anisaldehyde stain. After workup, organic phases were dried over Na_2SO_4/$MgSO_4$ and filtered before being concentrated under reduced pressure. ^1H and ^{13}C NMR spectra for the synthesized compounds were recorded at 298 K on an Agilent Technologies 400 MR spectrometer at 400 MHz or 100 MHz, respectively, or on Bruker Avance Neo spectrometers at 500/600 MHz or 125/150 MHz, respectively. Chemical shifts are reported in parts per million (ppm, δ) and referenced to the residual ^1H resonance of the solvent (CD_3OD δ 3.31) and the residual ^{13}C resonance of the solvent (CD_3OD δ 49.0). Splitting patterns are designated as follows: s (singlet), d (doublet), t (triplet), m (multiplet) and br (broad). Coupling constants (J) are listed in hertz (Hz). Preparative reversed-phase HPLC was performed on a Kromasil C8 column (250 × 21.2 mm, 5 μm) on a Gilson HPLC equipped with a Gilson 322 pump, a UV/Visible-156 detector and a 202 collector using acetonitrile-water gradients as eluents with a flow rate of 15 mL/min and detection at 210 or 254 nm. All tested compounds were purified by HPLC. ^1H NMR spectroscopy (500 MHz) and LC/MS were used to determine the purity of the compounds **30**, **65**, **68**, **78**, which all had a purity ≥95% (Supplementary Figures S16-S23).

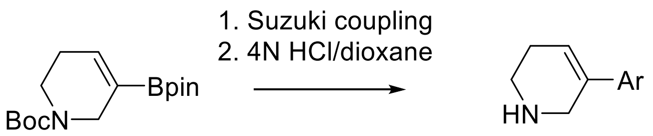

Procedure for Suzuki Coupling of 3-piperideine derivatives

Briefly, compounds **30**, **68** and **78** were synthesized starting from N-Boc protected pinacol borane precursor (scheme above) using Suzuki coupling with heteroaryl bromides followed by Boc-deprotection by 4N HCl in dioxane. General procedure: the mixture of the pinacol borane precursor (0.1 mmol, 1.3 eq, 31 mg), heteroaryl bromide (0.075 mmol, 1 eq), $PdCl_2dppf$ (2.5 mg, 0.05 eq), and Na_2CO_3 (16 mg, 2 eq) in dioxane: H_2O (3:1, v/v, 0.4 mL) was stirred at 100 °C for 2 hrs. After

cooling and removal of solvent, the residue was dissolved in DMSO, filtered and purified by HPLC using 20-100% acetonitrile in H_2O to afford the Boc-protected product. The later was dissolved in 4N HCl in dioxane (> 10 eq) and stirred at room temperature for 1 h. The solvent was then removed to afford desired product as a HCl salt.

Analytical data for compounds 30, 65, 68 and 78

Compound **30**

LC/MS (ESI+): calculated for $C_9H_{12}NO$ $(M+H)^+$: 150.1; found 149.9.
^1H NMR (500 MHz, CD_3OD) δ 7.50 (d, *J* = 1.8 Hz, 1H), 6.49 – 6.39 (m, 3H), 3.98 (q, *J* = 2.2 Hz, 2H), 3.37 (t, *J* = 6.2 Hz, 2H), 2.59 (dtt, *J* = 6.4, 4.5, 2.2 Hz, 2H). ^{13}C NMR (125 MHz, CD_3OD) δ 152.0, 143.9, 123.0, 119.8, 112.3, 106.7, 42.4, 41.7, 22.6. (Fig. S16-S17)

Compound **65**

LC/MS (ESI+): calculated for C10H14NO $(M+H)^+$: 164.2; found 164.0
^1H NMR (500 MHz, CD_3OD) δ 7.54 (d, *J* = 1.9 Hz, 1H), 6.51 (dd, *J* = 3.4, 1.9 Hz, 1H), 6.46 (d, *J* = 3.4 Hz, 1H), 3.95 (h, *J* = 2.0 Hz, 2H), 3.35 (t, *J* = 6.3 Hz, 2H), 2.54 (t, *J* = 6.6 Hz, 2H), 2.04 (t, *J* = 1.5 Hz, 3H). ^{13}C NMR (125 MHz, CD_3OD) δ 151.9, 143.2, 131.1, 117.0, 112.2, 110.2, 43.9, 41.8, 29.4, 21.1. (Fig. S18-S19)

Compound **68**

LC/MS (ESI+): calculated for $C_9H_{12}NO$ $(M+H)^+$: 150.1; found 149.9.
^1H NMR (500 MHz, CD_3OD) δ 7.62 (s, 1H), 7.51 (t, *J* = 1.7 Hz, 1H), 6.67 (dd, *J* = 1.9, 0.9 Hz, 1H), 6.25 (tt, *J* = 4.1, 1.8 Hz, 1H), 3.93 (q, *J* = 2.2 Hz, 2H), 3.36 (t, *J* = 6.2 Hz, 2H), 2.56 (tdt, *J* = 6.3, 4.3, 2.3 Hz, 2H). ^{13}C NMR (125 MHz, CD_3OD) δ 145.3, 139.6, 125.0, 124.4, 120.6, 108.0, 43.8, 41.7, 22.8. (Fig. S20-S21)

Compound **78**

The compound was obtained as formic acid salt after purification by HPLC using 0-40% acetonitrile in H_2O (H_2O + 0.1% formic acid).

LC/MS (ESI+): calculated for $C_{13}H_{14}NO$ $(M+H)^+$: 200.1; found 200.1.
^1H NMR (600 MHz, CD_3OD) δ 7.90 – 7.84 (m, 2H), 7.52 (d, *J* = 8.2 Hz, 1H), 7.39 – 7.34 (m, 1H), 7.34 – 7.29 (m, 1H), 6.59 (t, *J* = 4.1 Hz, 1H), 4.04 (s, 2H), 3.40 (t, *J* = 6.3 Hz, 2H), 2.65 (q, *J* = 5.9 Hz, 2H). ^{13}C NMR (150 MHz, CD_3OD) δ 157.2, 143.1, 126.3, 126.1, 124.9, 124.4, 123.2, 121.9, 120.0, 112.7, 44.3, 41.7, 23.3. (Fig. S22-S23)

Supplementary Figures

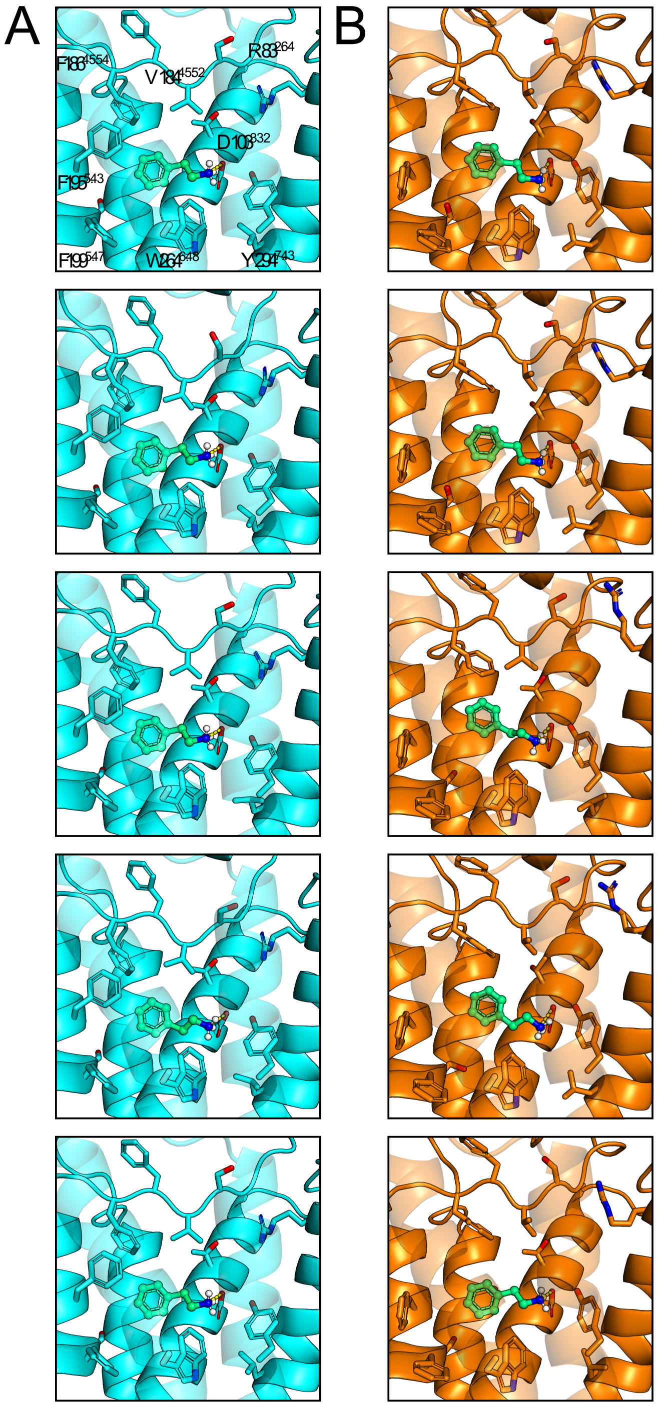

Fig. S1. Predicted binding modes of β-PEA in models used for prospective screens. Comparison of binding modes of β-PEA in (A) AlphaFold and (B) homology models. The receptor is shown as cyan (AlphaFold) and orange (homology model) ribbons. Ligands and key binding site residues are shown as sticks.

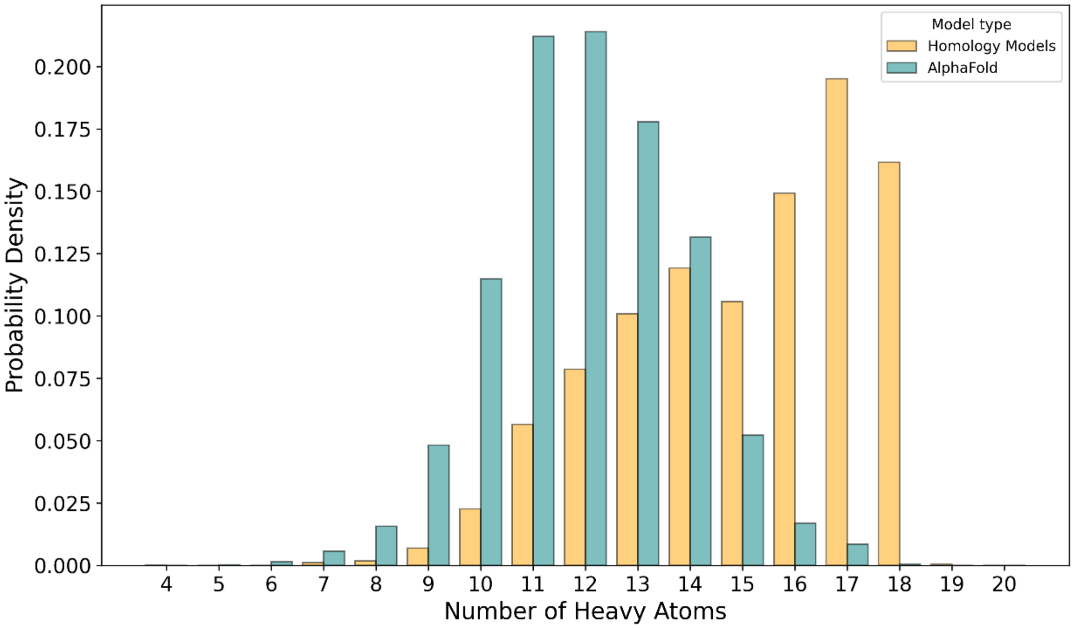

Fig. S2. Heavy atom distribution for top-ranked compounds from the docking screens.

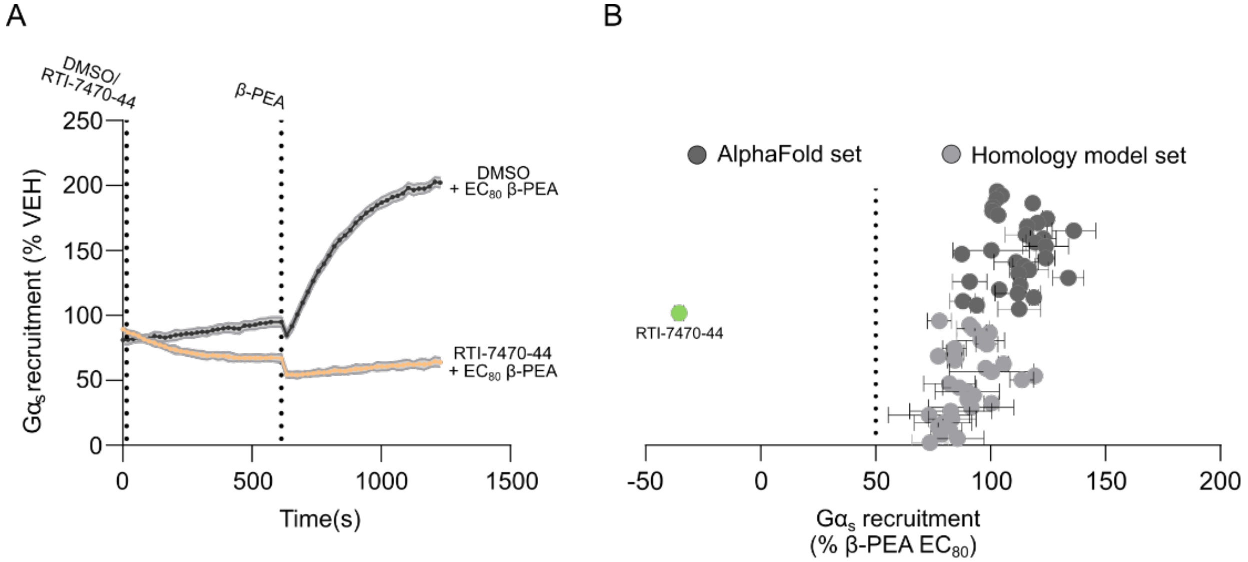

Fig. S3. Compound screening in antagonist mode. (A) Representative traces of $G\alpha_s$ recruitment to TAAR1 run in antagonist mode with RTI-7470-44 or DMSO treatment for 10-minutes prior to stimulation with an EC_{80} concentration of β-PEA. Data are from technical duplicates with error bars in S.D shown as a gray background. Data are normalized to vehicle at time = 0. (B) Compounds or RTI-7470-44 were tested at 20 µM in technical duplicates. Error bars represent S.D. Data are normalized to $G\alpha_s$ recruitment elicited by Vehicle-β-PEA at the end of the 10-minute stimulation period.

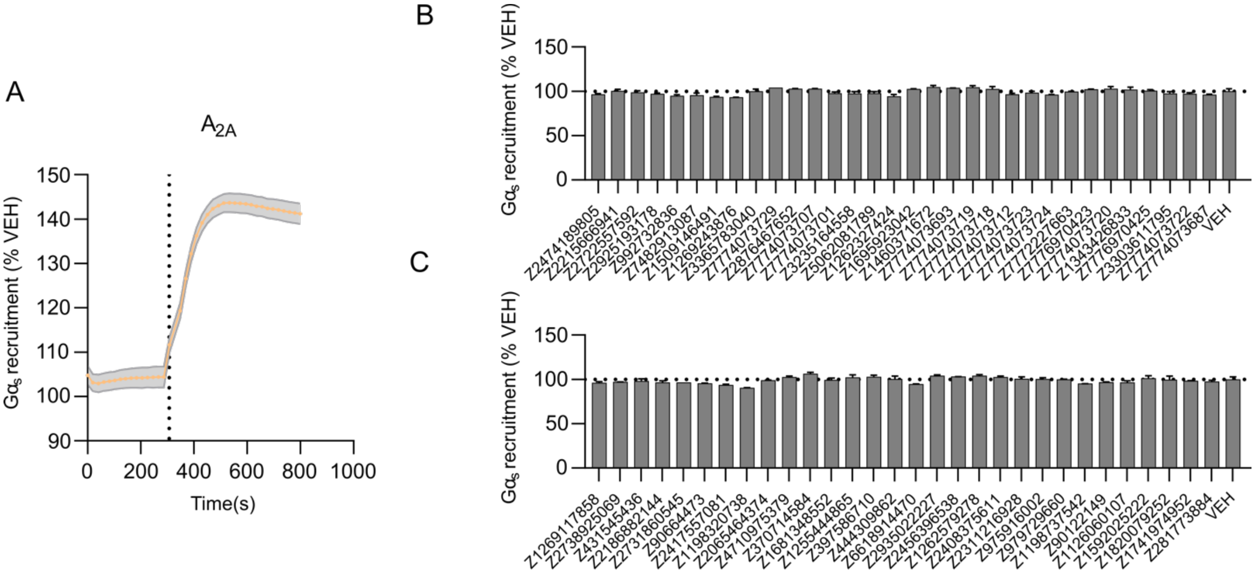

Fig. S4. Counter screen at the A_{2A}R. (A) Validation of functional LgBiT-Gα_s recruitment to SmBiT tagged A_{2A}R with cyclopentyladenosine (CPA, 20 μM) stimulation, normalized to vehicle. Dashed line indicates time of CPA addition. (B) Homology modeling and (C) AlphaFold hits were counter-screened using this setup. Gα_s recruitment for each compound is normalized to vehicle (100%). Each bar represents 2-4 technical replicates ± S.D.

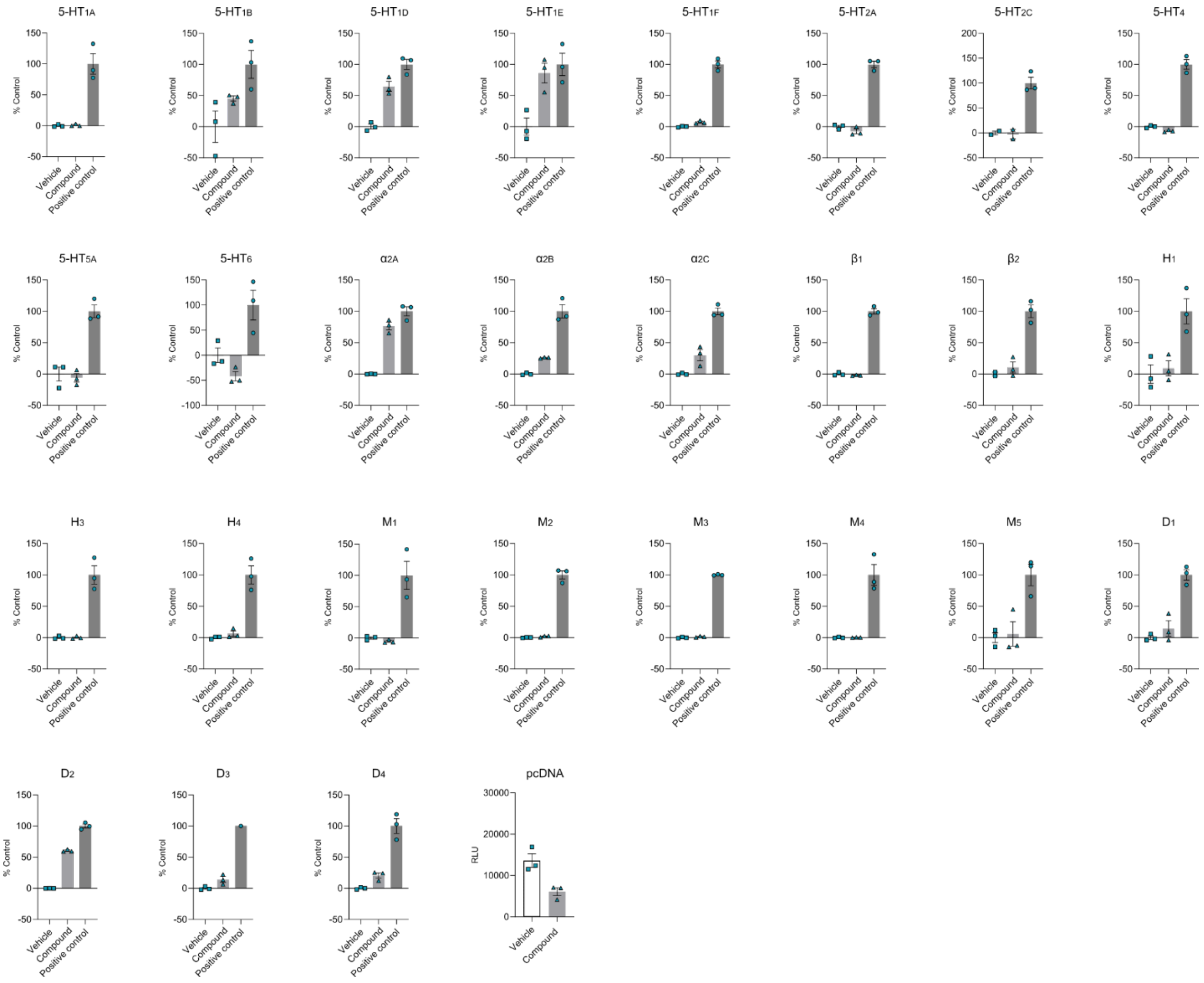

Fig. S5. PRESTO-TANGO aminergic GPCR screening for Ulotaront. Each data point is normalized to the mean of the positive control. Error bars represents mean ± SEM.

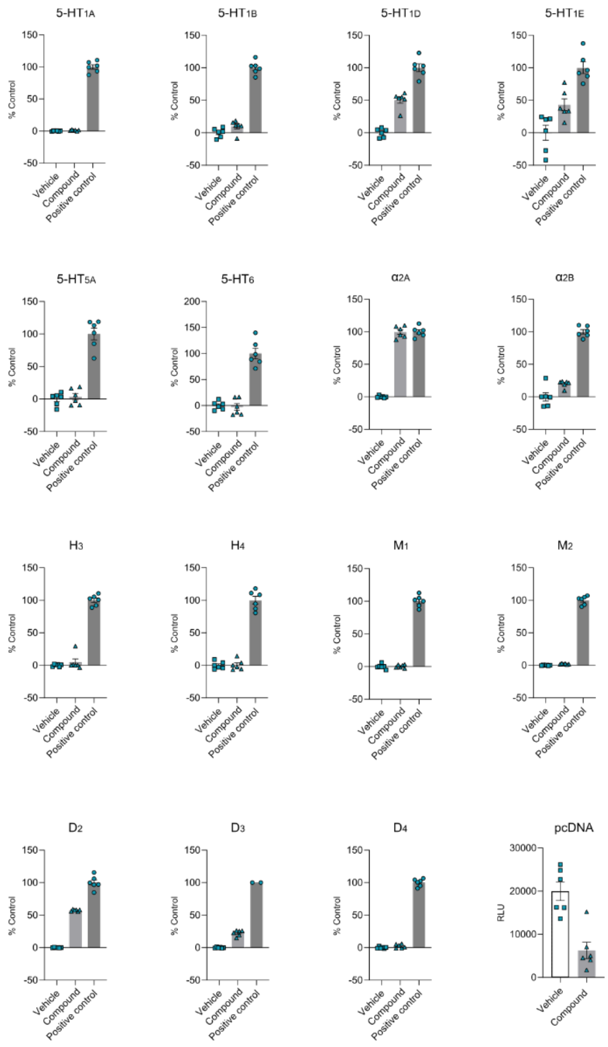

Fig. S6. PRESTO-TANGO aminergic GPCR screening for compound 65. Each data point is normalized to the mean of the positive control. Error bars represents mean ± SEM.

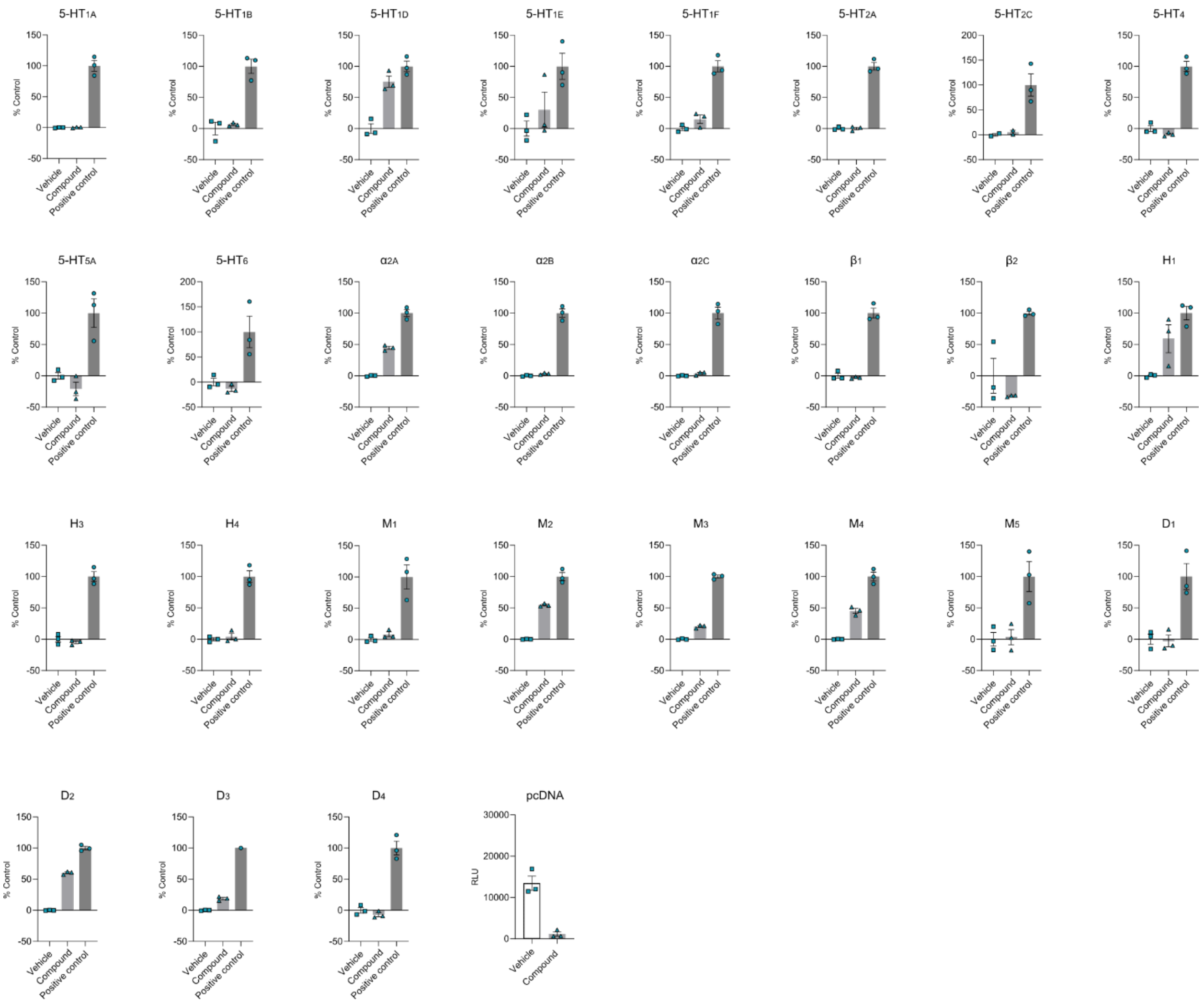

Fig. S7. PRESTO-TANGO aminergic GPCR screening for compound 30. Each data point is normalized to the mean of the positive control. Error bars represents mean ± SEM.

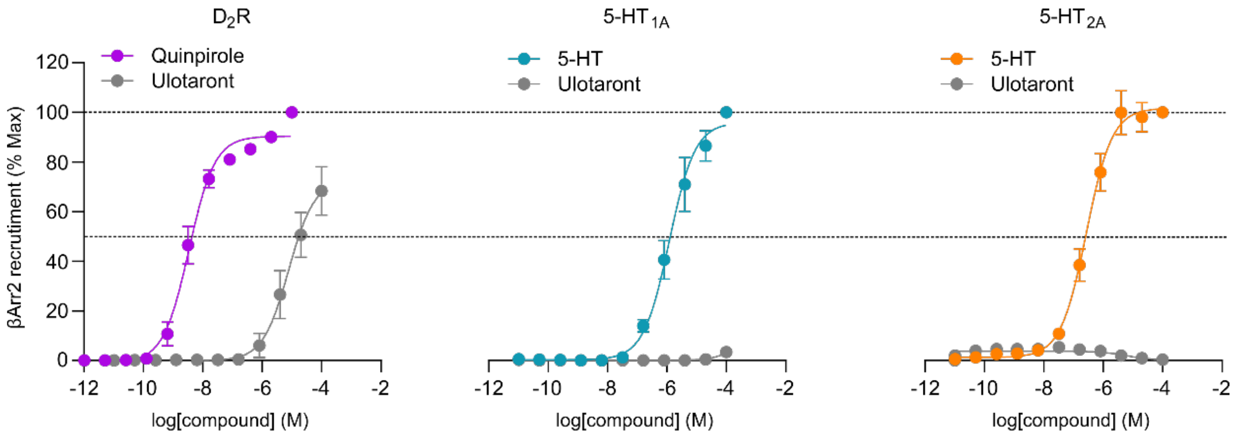

Fig. S8. Concentration response curves for Ulotaront at the D_2, 5-HT_{1A} and 5-HT_{2A} receptors using the PRESTO-TANGO assay. Each data point represents mean ± SEM from three independent experiments.

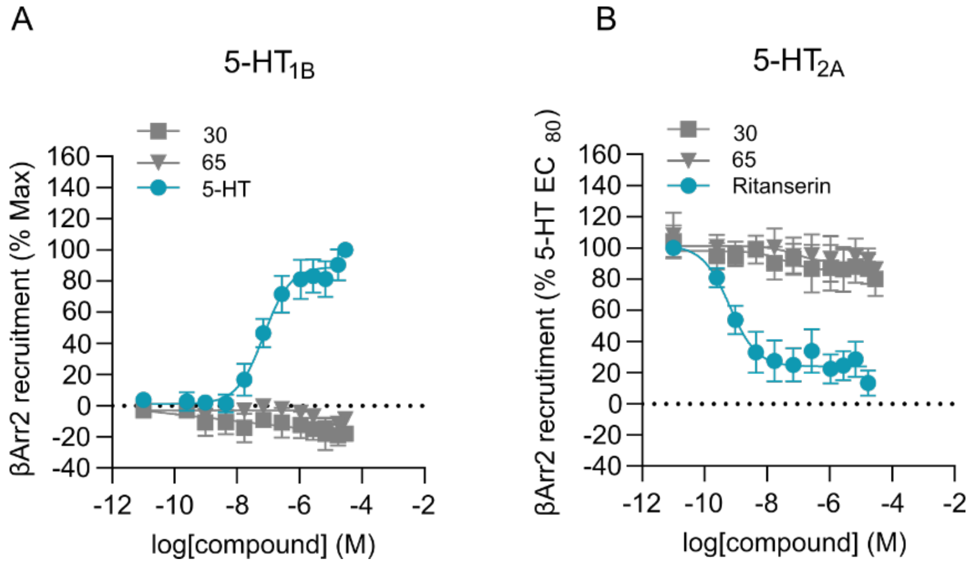

Fig. S9. Concentration response curves for compounds 30 and 65 at the 5-HT$_{1B}$ and 5-HT$_{2A}$ receptors using the PRESTO-TANGO assay. (A) Concentration-response profiles of compounds **30** and **65** at the 5-HT1$_{B}$ and (B) 5-HT$_{2A}$ receptors, assessed with PRESTO-TANGO. Each data point represents mean ± SEM from three independent experiments.

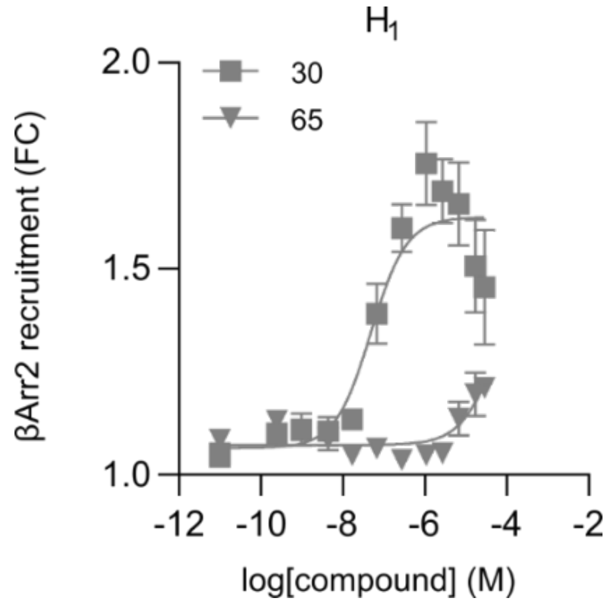

Fig. S10. Concentration response curves for compounds 30 and 65 at the H_1 receptor using the PRESTO-TANGO assay. Each data point represents mean ± SEM from three independent experiments and are normalized to vehicle only controls.

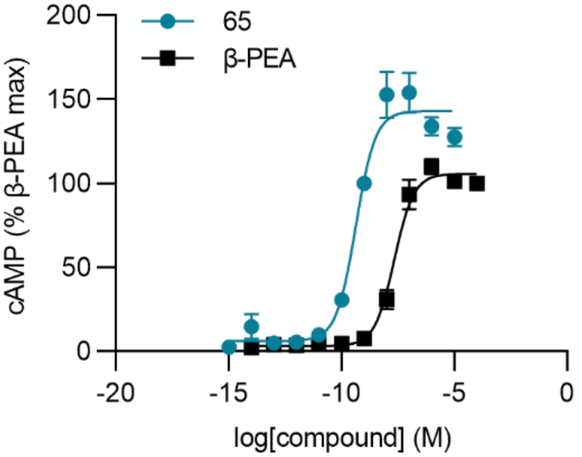

Fig. S11. Evaluation of compound 65 in cAMP assays. Compound **65** (teal) or β-PEA (black) were evaluated for their ability to increase cAMP in hTAAR1 transfected Expi293F cells. Each dot represents mean ± SEM from 2-3 independent experiments with 6 technical replicates each.

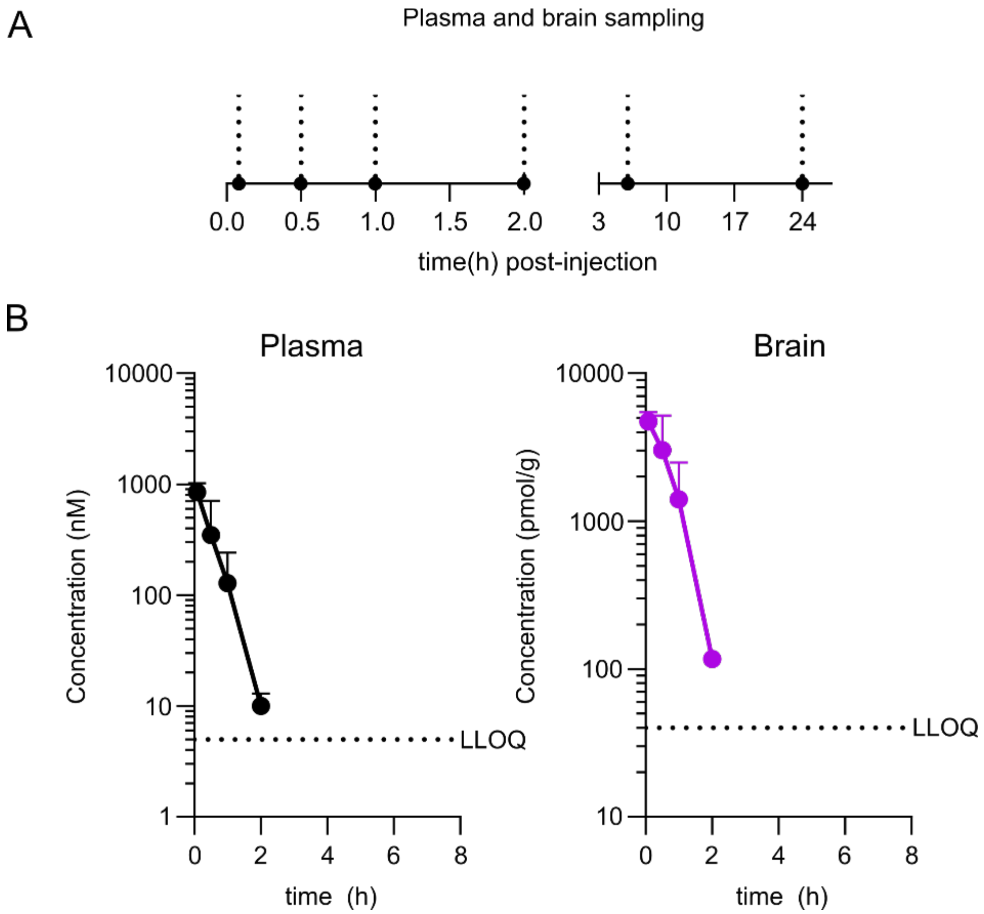

Fig. S12. Pharmacokinetic profile of compound 65 in mice. (A) Overview of sample collection time-points. (B) Concentration-time curves of compound **65** in plasma and brain after i.p. administration at 1 mg/kg to male C57BL/6 mice. Data are represented as means ± SD (n = 3). Horizontal dotted lines show lower limit of quantification (LLOQ) for the quantification in plasma (5 nM) and brain (40 pmol/g brain), respectively.

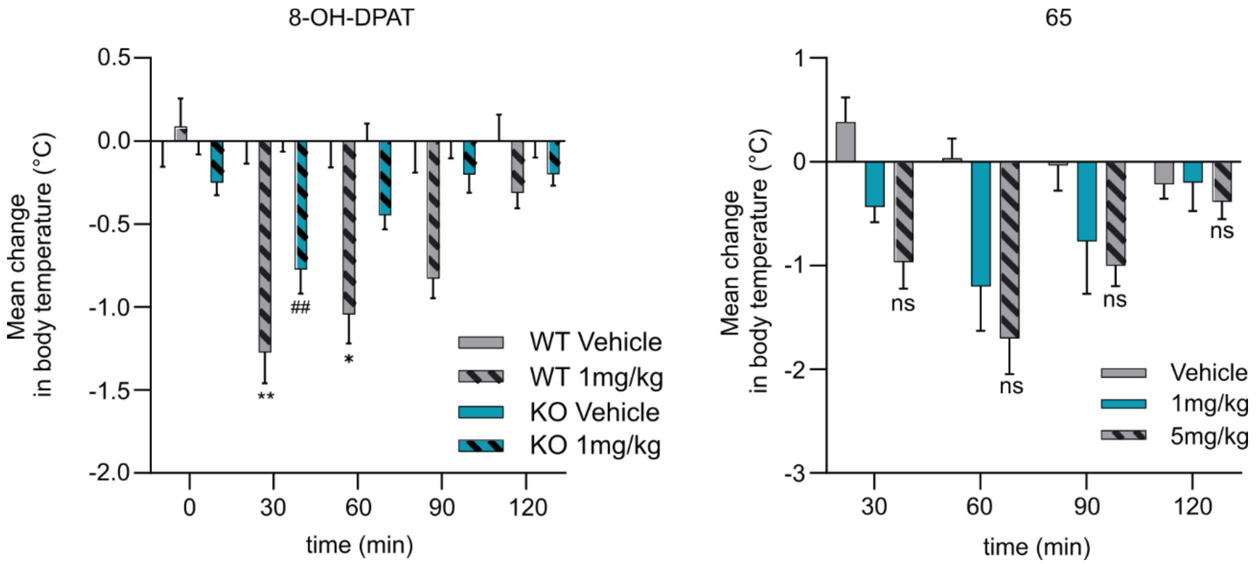

Fig. S13. Further CBT measurements in WT and TAAR1-KO mice. (A) Responsiveness of WT and TAAR1-KO mice to 1 mg/kg 8-OH-DPAT, a 5-HT_{1A}R agonist (n=5-7 mice per condition, **p<.005, *p<.05 WT 1 mg/kg vs. WT Vehicle, ##p<.005 TAAR1-KO 1 mg/kg vs TAAR1-KO Vehicle, 2-way ANOVA, Bonferroni's multiple comparison test.) (B) TAAR1-WT mice (n=6 for all groups) were injected with compound **65** and CBT measured in 30-min intervals. (ns: not significant, 5 mg/kg vs. 1 mg/kg) based on 2-way ANOVA, Bonferroni's multiple comparison test) Error bars represent mean ± SEM.

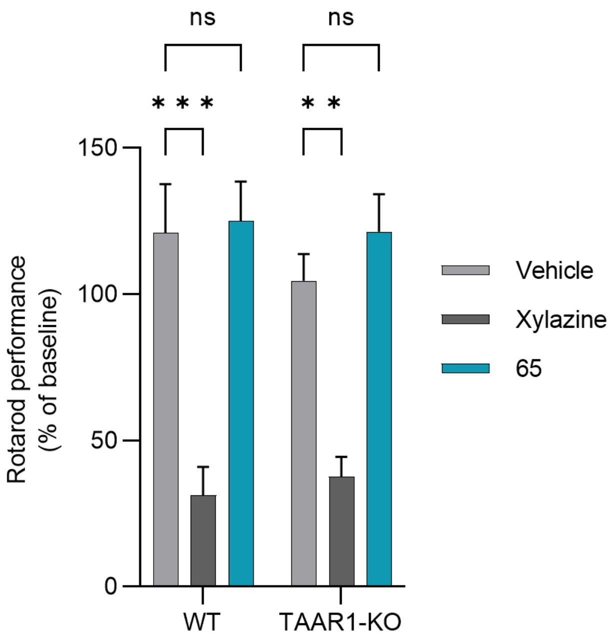

Fig. S14. Evaluation of compound 65 impact on rotarod performance. Vehicle, xylazine or **65** impact on rotarod performance in WT (n=8) and TAAR1-KO mice (n=6). Performance was normalized to that of the baseline read prior to injection with compound or vehicle. **p<.005, ***p<.001 (**65** or xylazine vs. vehicle) based on 2-way ANOVA, Bonferroni's multiple comparison test.

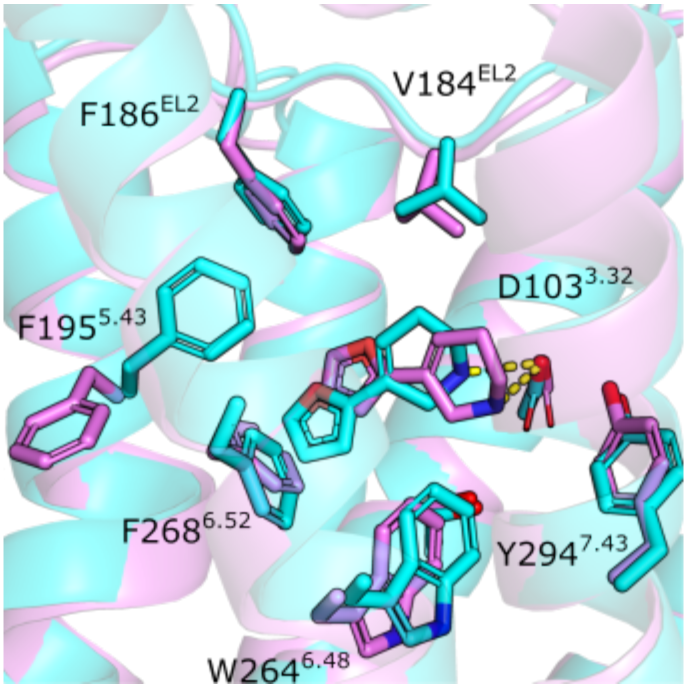

Fig. S15. Predicted binding mode of compound 30 in the AlphaFold and experimental structure. Comparison of an AlphaFold model of TAAR1 (cyan) to the cryo-EM structure with PDB accession code 8W89 (violet). The receptor is shown as a cartoon. Binding site side chains and the ligand are shown as sticks. Polar interactions are shown as yellow dashed lines.

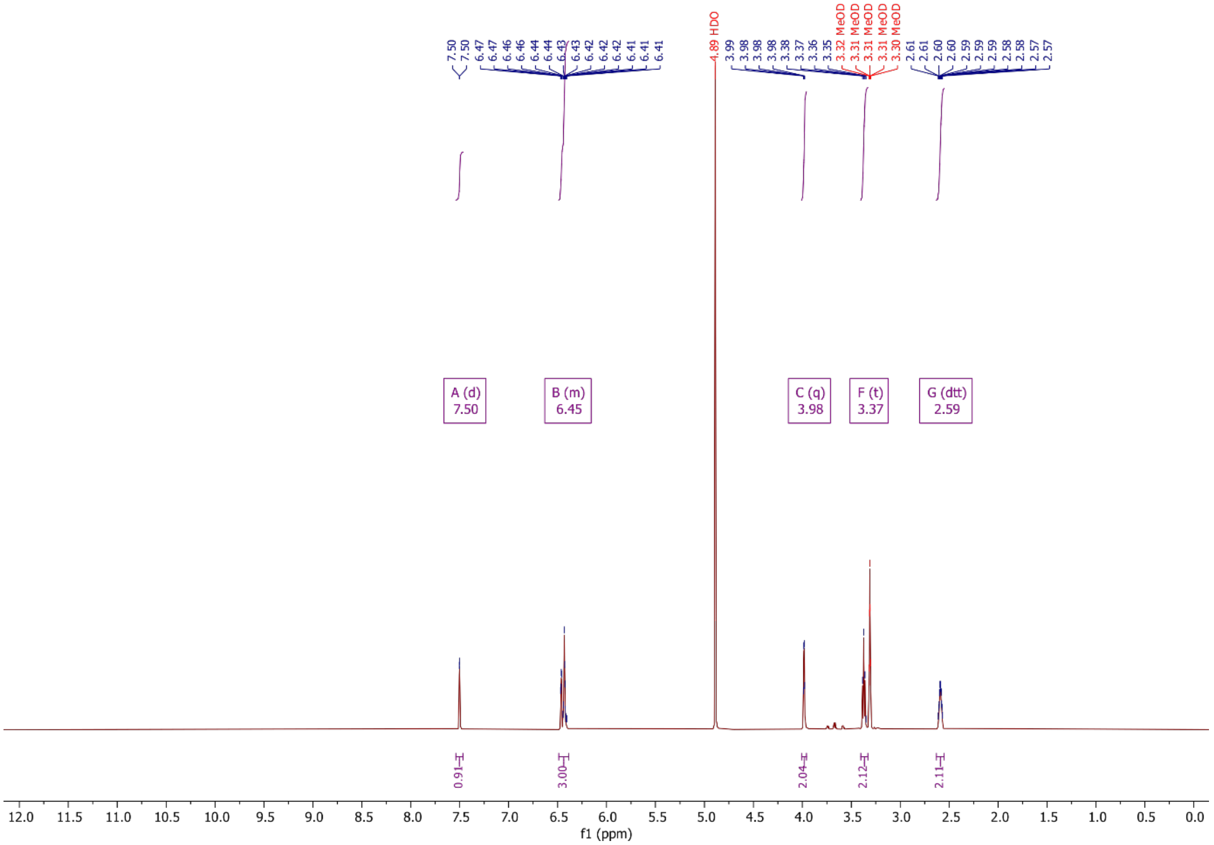

Fig. S16. Compound **30** ^{1}H NMR spectra.

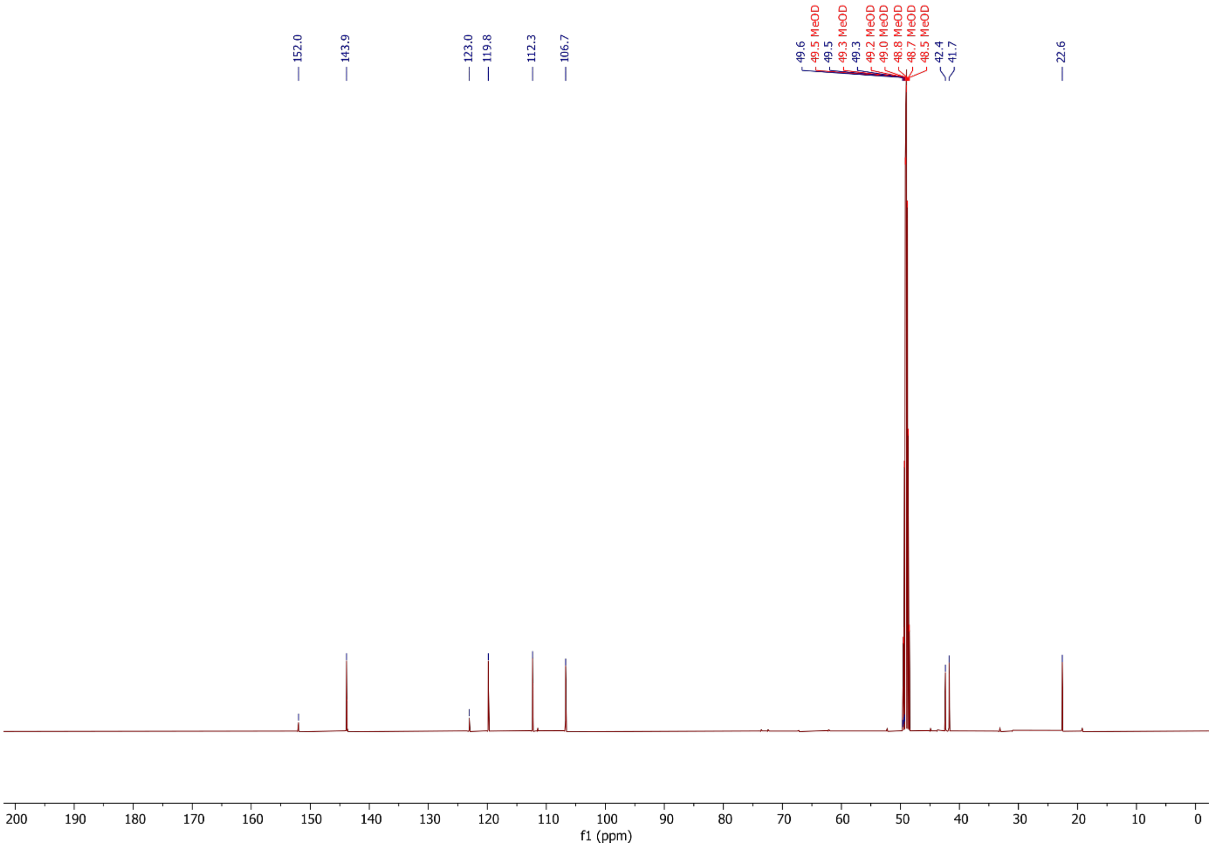

Fig. S17. Compound **30** ^{13}C NMR spectra.

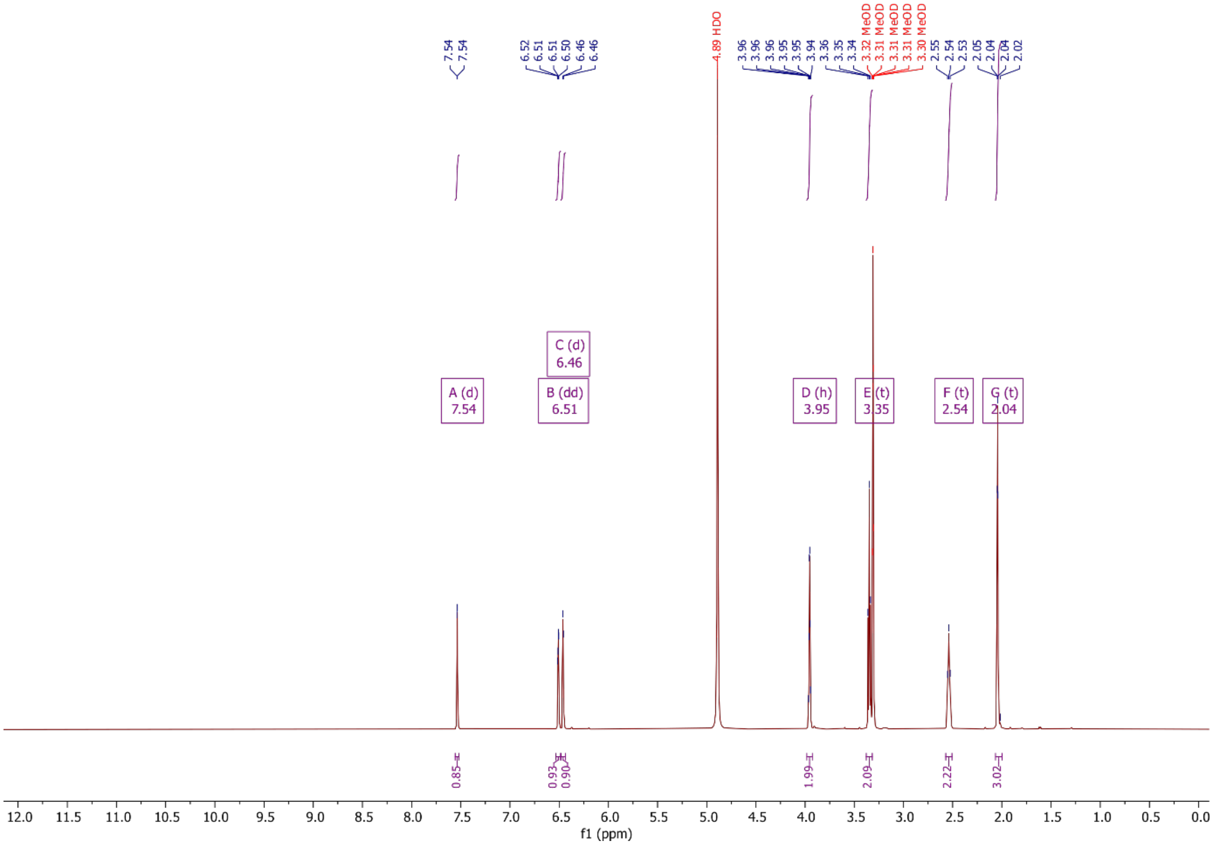

Fig. S18. Compound **65** ^{1}H NMR spectra.

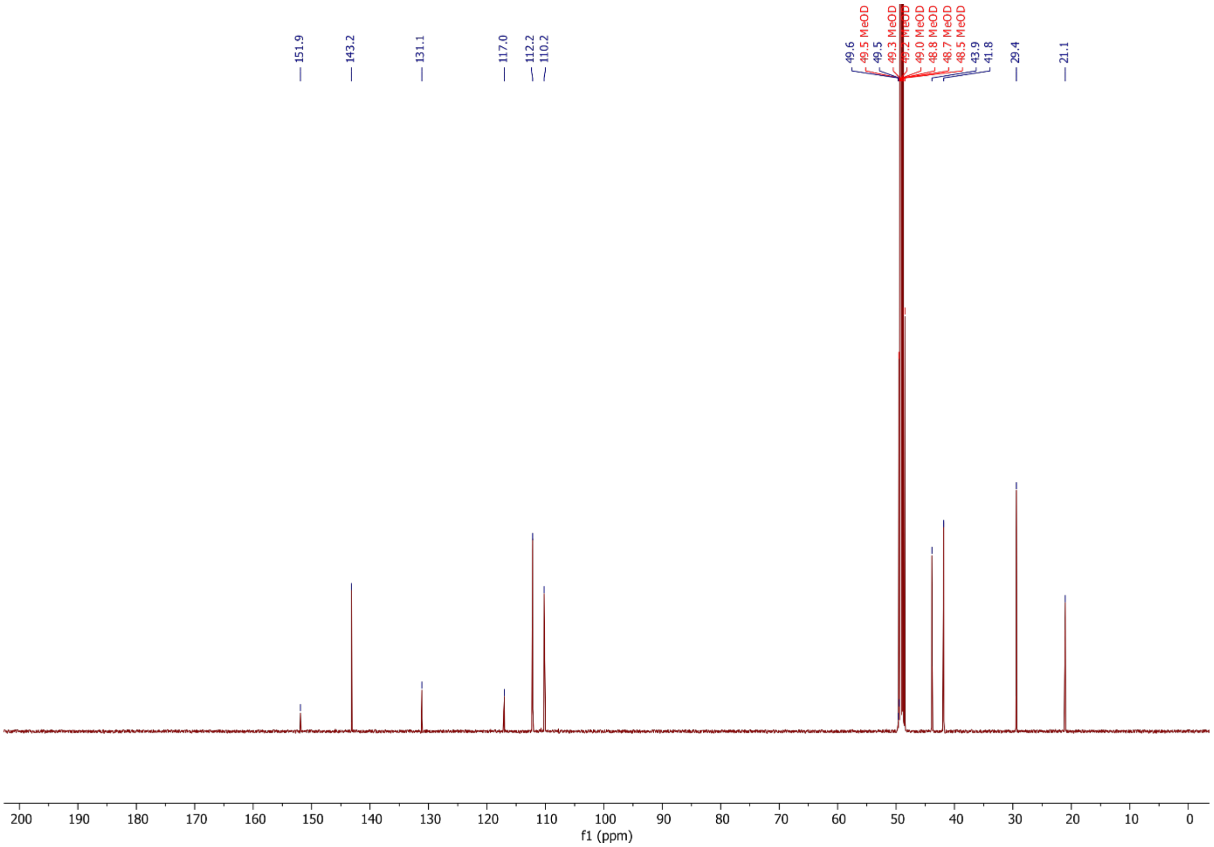

Fig. S19. Compound **65** ^{13}C NMR spectra.

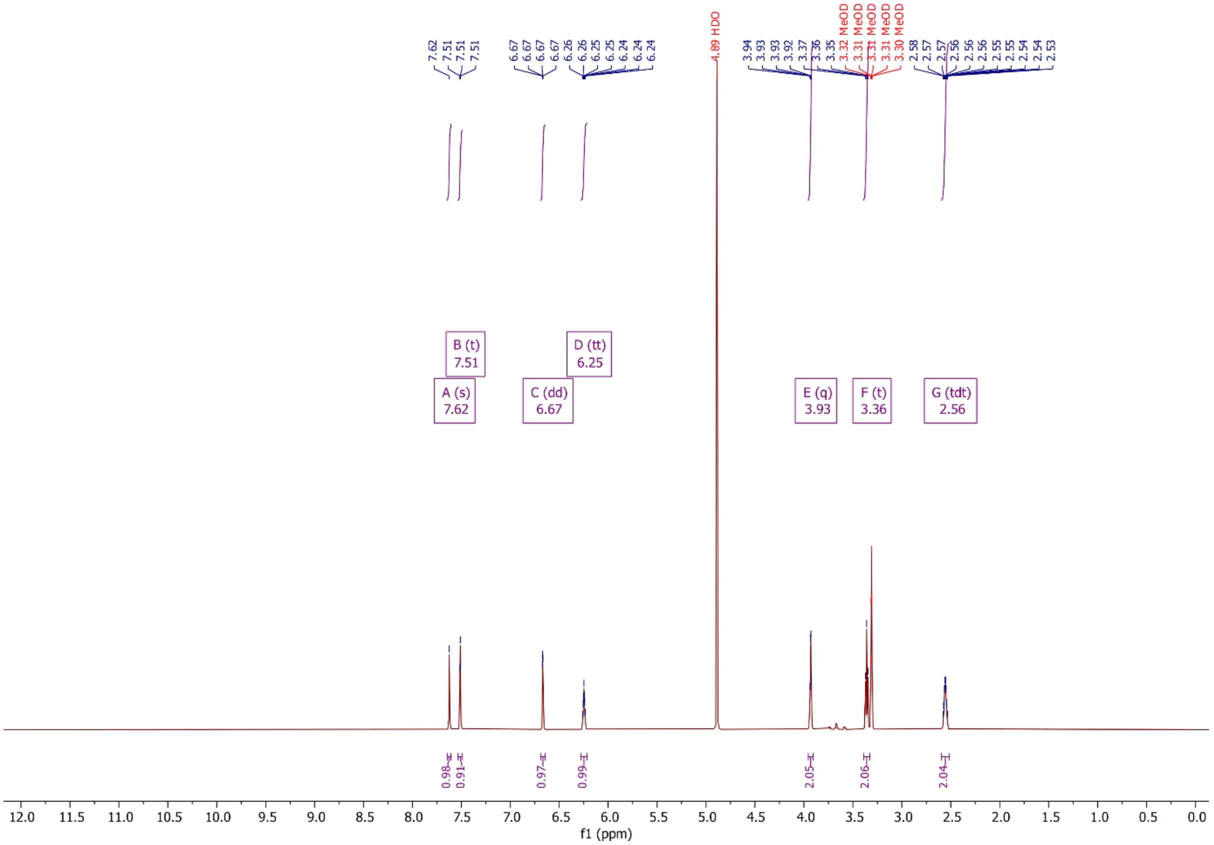

Fig. S20. Compound **68** ^{1}H NMR spectra.

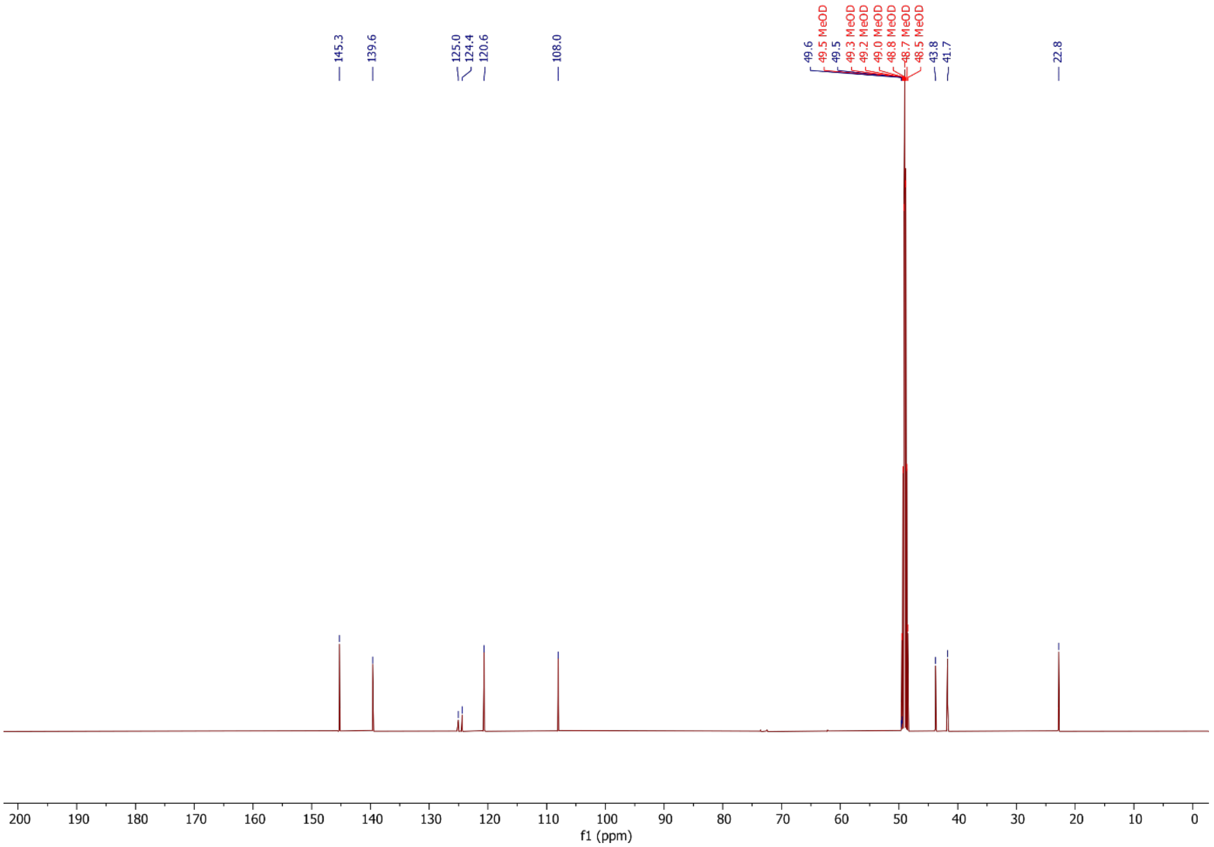

Fig. S21. Compound **68** ^{13}C NMR spectra.

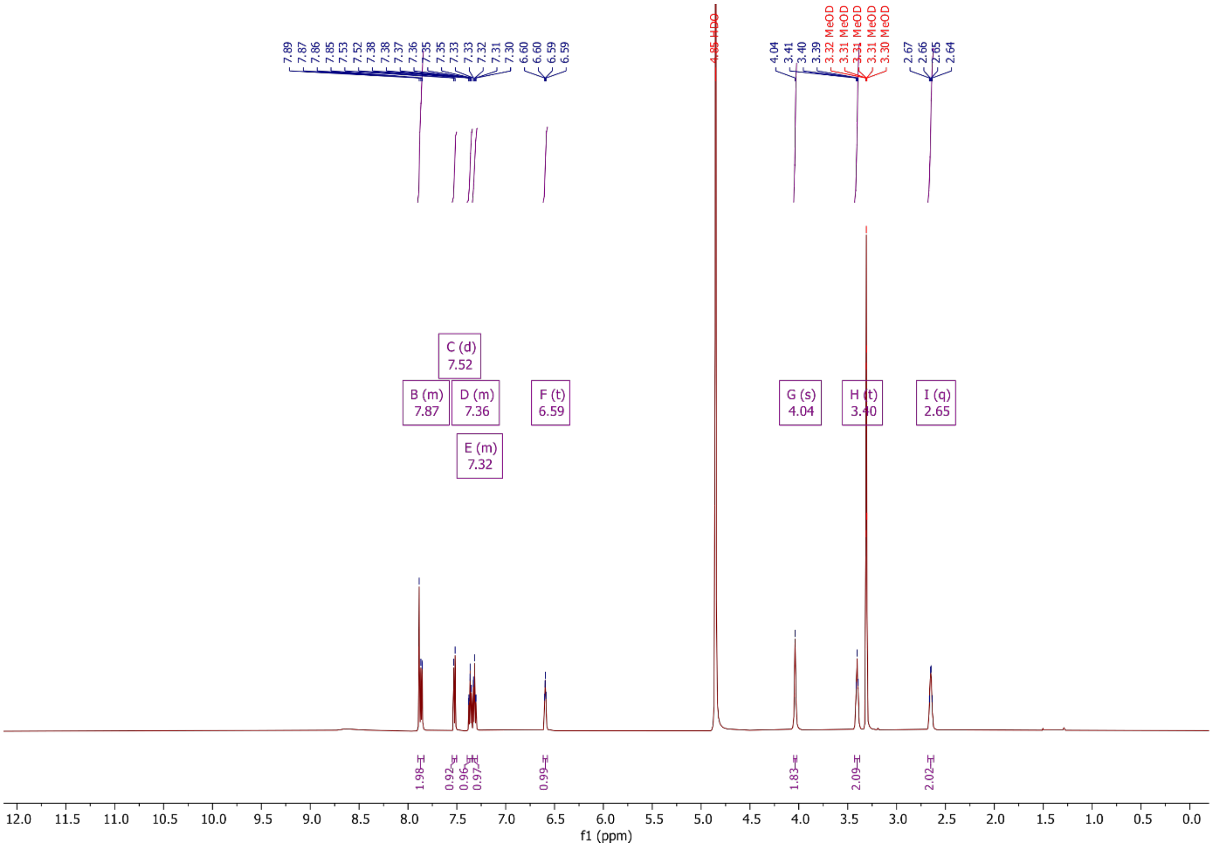

Fig. S22. Compound **78** ^{1}H NMR spectra.

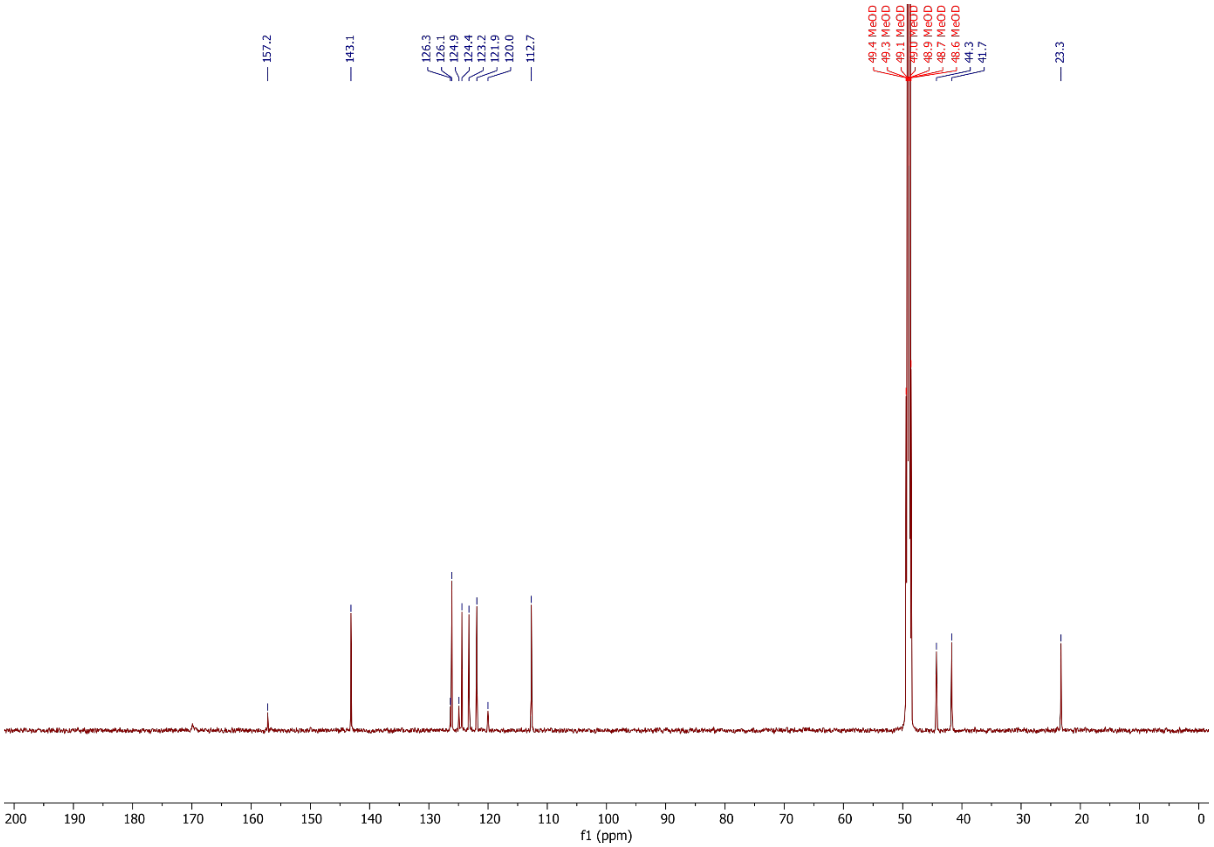

Fig. S23. Compound **78** ^{13}C NMR spectra.

Supplementary Tables

Table S1. Homology modelling templates for TAAR1 and ligand enrichment.

PDB code	Gene Name	Co-crystalized ligand	TM region sequence identity (%)	Homology model ligand enrichment[a]	
				Maximal LogAUC	Median LogAUC
7bu6	ADRB1	Norepinephrine	35%	23.7	15.0
4ldo	ADRB2	Adrenaline	33%	23.5	11.8
7ljd	DRD1	Dopamine	37%	20.6	13.2
7exd	HTR1F	Lasmiditan	35%	19.0	8.6

[a] Ligand enrichment was assessed using the adjusted LogAUC based on docking of known TAAR1 agonists and property-matched decoys to 250 models/template.

Table S2. TAAR1 models used in prospective virtual screening.

Model	Ligand enrichment	
	LogAUC	EF1%
AlphaFold model 1	28.6	19.5
AlphaFold model 2	32.0	22.0
AlphaFold model 3	28.7	20.9
AlphaFold model 4	27.4	16.7
AlphaFold model 5	28.5	17.3
Homology model 1	26.3	15.4
Homology model 2	25.8	13.0
Homology model 3	27.4	14.8
Homology model 4	26.0	14.9
Homology model 5	25.0	11.3

Table S3A. Compounds predicted based on virtual screens using AlphaFold models.

Cmpd	ZINC database code	Vendor code (Enamine)	T_c[a]	Docking rank[b]	E_{max}[c]
**1**	ZINC000058235688	Z1198320738	0.22	2132	1.2
**2**	ZINC000003732261	Z281773884	0.29	1639	1.3
**3**	ZINC000074309947	Z2408375611	0.16	3474	2.3
**4**	ZINC000004203917	Z1741974952	0.19	3932	4.4
**5**	ZINC000032625181	Z444309862	0.24	3531	8.5
**6**	ZINC000004206577	Z90122149	0.21	2488	8.8
**7**	ZINC000003705358	Z1126060107	0.22	3285	14.7
**8**	ZINC000090225705	Z1820079252	0.35	3110	14.9
**9**	ZINC000035762860	Z431545436	0.20	1785	19.1
**10**	ZINC000051783779	Z975916002	0.17	2671	27.1
**11**	ZINC000004271676	Z1262579278	0.17	1439	40.4
**12**	ZINC000012370199	Z1255444865	0.22	1747	45.7
**13**	ZINC000082851814	Z2738925069	0.22	2938	51.8
**14**	ZINC000082405087	Z1198737542	0.23	1304	54.3
**15**	ZINC000026515978	Z370714584	0.22	311	57.5
**16**	ZINC000003705343	Z90664473	0.23	4001	61.9
**17**	ZINC000020221786	Z1269117858	0.22	1017	67.8
**18**	ZINC000629717281	Z6618914470	0.20	84	68.7
**19**	ZINC000021298511	Z2417557081	0.21	573	72.8
**20**	ZINC000082351154	Z2731860545	0.22	151	74.5
**21**	ZINC000685939341	Z2935022227	0.20	555	75.3
**22**	ZINC000012956959	Z4710975379	0.27	3272	78.5
**23**	ZINC000075546278	Z2456396538	0.20	1528	79.2
**24**	ZINC000033638506	Z1592025222	0.34	668	81.8
**25**	ZINC000011632015	Z979729660	0.18	717	95.3
**26**	ZINC000117868735	Z2186882144	0.36	3040	95.5
**27**	ZINC000036965089	Z397586710	0.20	3060	98.7
**28**	ZINC000238493985	Z1681348552	0.18	187	102.6
**29**	ZINC000082326504	Z2311216928	0.15	381	105.7
**30**	ZINC000225517359	Z2065464374	0.13	963	112.5

[a] Maximal Tanimoto similarity coefficient (Tc) between the compound and all ChEMBL ligands of TAAR1 (activity ≥5). [b] Ranking in the chemical library prior to clustering. All selected compounds were among the top 2000 cluster centers.[c] E_{max} values are relative to the maximal effect of the reference agonist β-PEA (20 μM).

Table S3B. Compounds predicted based on virtual screens using homology models.

Cmpd	ZINC database code	Vendor code (Enamine)	T_c[a]	Docking rank[b]	E_{max}[c]
**31**	ZINC000012370365	Z7774073729	0.28	5296	-2.6
**32**	ZINC000000127291	Z3235164558	0.24	2682	-1.1
**33**	ZINC000115000061	Z1269243876	0.29	708	0.6
**34**	ZINC000159582108	Z7774073687	0.26	1573	0.7
**35**	ZINC000924627897	Z7482913087	0.22	1470	0.9
**36**	ZINC000381310833	Z1343426833	0.39	3493	1.3
**37**	ZINC000086251164	Z7774073724	0.26	2774	2.7
**38**	ZINC000924569626	Z2925193178	0.31	896	3.5
**39**	ZINC000053294456	Z992732836	0.24	4274	3.9
**40**	ZINC001308417590	Z7774073723	0.39	2345	5.8
**41**	ZINC000005424656	Z7774073701	0.32	1280	7.3
**42**	ZINC000527725438	Z7774073693	0.16	3577	7.6
**43**	ZINC000649833907	Z2474189805	0.20	4854	7.6
**44**	ZINC000863565390	Z2722557592	0.17	3558	10.0
**45**	ZINC000090397967	Z7772227663	0.22	3571	10.7
**46**	ZINC000304250846	Z5062081789	0.16	4860	18.8
**47**	ZINC001474299771	Z3365783040	0.21	2106	21.0
**48**	ZINC000631035035	Z7774073712	0.20	2567	25.9
**49**	ZINC000001676213	Z1509146491	0.32	910	27.1
**50**	ZINC000886312094	Z7774073719	0.34	829	29.0
**51**	ZINC000849013551	Z7776970423	0.22	2790	41.2
**52**	ZINC000144288155	Z2215666941	0.27	5480	44.5
**53**	ZINC000036900188	Z7774073720	0.37	847	45.1
**54**	ZINC001243109116	Z7776970425	0.15	5422	47.9
**55**	ZINC000082442186	Z3303611795	0.22	3450	48.7
**56**	ZINC000036167379	Z7774073707	0.30	2811	63.0
**57**	ZINC000021964096	Z1262327424	0.20	290	73.2
**58**	ZINC000178158325	Z1460371672	0.33	3350	78.4
**59**	ZINC000069049545	Z7774073718	0.23	550	78.8
**60**	ZINC000336226092	Z7774073722	0.21	1877	79.9
**61**	ZINC000054764984	Z2876467652	0.39	1359	80.3
**62**	ZINC000038540946	Z1695923042	0.22	3728	107.4

[a] Maximal Tanimoto similarity coefficient (Tc) between the compound and all ChEMBL ligands of TAAR1 (activity ≥5). [b] Ranking in the chemical library prior to clustering. All selected compounds were among the top 2000 cluster centers.[c] E_{max} values are relative to the maximal effect of the reference agonist β-PEA (20 μM).

Table S4. Analogs of hits from the AlphaFold docking screen.

Cmpd	Structure	pEC_{50}[a]	Cmpd	Structure	pEC_{50}[a]
**63**	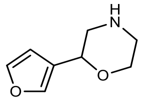	6.7	**73**	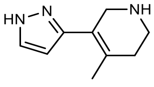	6.2
**64**	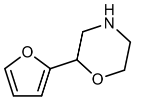	6.4	**74**	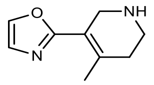	6.2
**65**	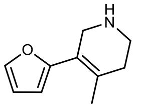	7.6	**75**	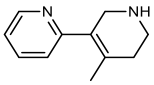	6.3
**66**	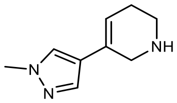	5.7	**76**	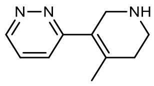	4.3
**67**	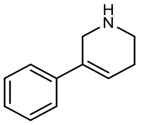	7.6	**77**	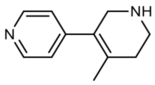	5.1
**68**	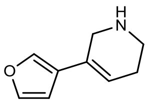	7.5	**78**	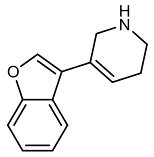	5.7
**69**	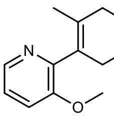	5.5			
**70**	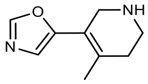	5.8			
**71**	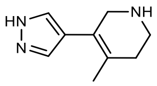	5.8			
**72**	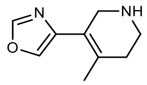	6.2			

[a] pEC_{50} values at TAAR1 using a G protein recruitment assay.

Table S5. Compounds used as positive controls for PRESTO-TANGO aminergic receptor screening.

Control	Target
2-Pyridylethylamine dihydrochloride	H_1
Amthamine dihydrobromide	H_2
IMMEPIP 2HBr	H_3
4-Methylhistamine dihydrochloride	H_4
Racepinephrine HCl	Adrenergic receptors
Serotonin hydrochloride	5-HT receptors
(-)-Quinpirole hydrochloride	D_2
Pramipexole	D_3
Dihydrexidine hydrochloride	D_1
Carbachol	Muscarinic receptors
Rotigotine	D_5

Table S6. ANOVA and mixed model statistical testing details.

Figure	Type	Corrections	ANOVA table	SS	DF	MS	F (DFn, DFd)	P value
**4C**	RM	Geisser-Greenhouse	Time x Treatment	8,191	12	0,6826	F (12, 92) = 5,279	P<,001
(WT)			Time	10,83	4	2,708	F (2,388, 54,93) = 20,94	P<,001
**4E**	RM		Pre-pulse intensity	10200	2	5100	F (2, 12) = 55,41	P<,001
(WT)			Treatment	889,4	2	444,7	F (2, 12) = 5,642	P=,019
(KO)			Pre-pulse intensity	17336	2	8668	F (2, 14) = 121,5	P<,001
			Treatment	2253	2	1126	F (2, 14) = 9,561	P=,002
**4F**	RM		Genotype x Treatment	259,4	2	129,7	F (2, 26) = 3,898	P=,033
			Treatment	757,7	2	378,8	F (2, 26) = 11,39	P<,001
**4H**	RM		Treatment	3E+06	2	2E+06	F (2, 36) = 6,866	P=,003
**4J**	RM		Genotype x Treatment	20280	2	10140	F (2, 36) = 3,455	P=,042
			Treatment	455387	2	227693	F (2, 36) = 77,58	P<,001
**SI CBT**	RM	Geisser-Greenhouse						
(8-OH DPAT)			Time x Treatment	5,179	12	0,4316	F (12, 76) = 7,984	P<,001
			Time	3,197	4	0,7994	F (1,823, 34,63) = 14,79	P<,001
			Treatment	8,885	3	2,962	F (3, 19) = 7,641	P=,002
(comp. 65)			Time x Treatment	4,507	6	0,7512	F (6, 45) = 4,130	P=,002
			Time	5,246	3	1,749	F (2,390, 35,85) = 9,614	P<,001
			Treatment	13,77	2	6,884	F (2, 15) = 4,968	P=,022
**SI Rotarod**	Ordinary		Treatment	65888	2	32944	F (2, 37) = 29,03	P<,001
	RM: Repeated Measures							

Table S7. Comparison between TAAR1 cryo-EM structures and computational models.

Region	Residues	RMSD (Å)[a]	
		AlphaFold models	Homology models
Extracellular TM region (Cα)[a]	Extracellular TM1-7[b]	0.7	1.1
Extracellular TM1 (Cα)	23-33	0.9	1.4
Extracellular TM2 (Cα)	72-84	0.6	1.0
Extracellular TM3 (Cα)	95-108	0.8	0.6
Extracellular TM4 (Cα)	150-161	0.8	1.0
Extracellular TM5 (Cα)	188-198	0.9	1.6
Extracellular TM6 (Cα)	264-276	0.9	0.9
Extracellular TM7 (Cα)	284-295	1.0	1.2
Extracellular loop 1 (Cα)	85-94	1.3	1.4
Extracellular loop 2 (Cα)	162-187	2.9	4.8
Extracellular loop 2 (binding site, Cα)	183-187	1.0	1.5
Extracellular loop 3 (Cα)	277-283	1.5	2.8
Extracellular loops (Cα)	85-94, 162-187, 277-283	2.8	4.1
Orthosteric binding site (Cα)	Residues within 5 Å of β-PEA[c]	0.7	0.9
Orthosteric binding site (side chain)	Residues within 5 Å of β-PEA[c]	1.4	1.6
Intracellular TM region (Cα)	Intracellular TM1-7[d]	2.5	1.0

[a] The structures were aligned based on the extracellular TM region, followed by calculations of the average RMSD values for different selections of residues. 13 cryo-EM structures (8JLN, 8JLO, 8JLP, 8JLQ, 8JLR, 8JSO, 8UHB, 8W87, 8W88, 8W89, 8W8A, 8WC8 and 8WCA) and five predicted structures (AlphaFold or homology models) were used in the calculations. The lowest value (*i.e.* most accurate model) is marked in green.

[b] The combination of all extracellular TM residues (TM1 to TM7).

[c] Residues 103, 104, 107, 108, 184, 186, 194, 198, 264, 267, 268, 271, 290, and 294. The binding site residues were identified using the receptor structure with PDB code: 8W89.

[d] Residues 34-49, 58-71, 109-123, 137-149, 199-218, 247-263, and 296-306.
